# Supplementary material for: Interpretable genotype-to-phenotype classifiers with performance guarantees
Source: Sci Rep. 2019 Mar 11;9:4071. doi: 10.1038/s41598-019-40561-2 (PMC6411721; doi:10.1038/s41598-019-40561-2)
Supplement: Supplementary file 1 — Supplementary information [file 41598_2019_40561_MOESM1_ESM.pdf]

Interpretable genotype-to-phenotype classifiers with performance guarantees

Supplementary information

Alexandre Drouin[1,2,*], Gaël Letarte[1,2], Frédéric Raymond[3,4], Mario Marchand[1,2], Jacques Corbeil[2,5], and François Laviolette[1,2]

[1]Department of Computer Science and Software Engineering, Université Laval, Quebec, Canada
[2]Big Data Research Centre, Université Laval, Quebec, Canada
[3]School of nutrition, Université Laval, Quebec, Canada
[4]Institute of Nutrition and Functional Foods, Université Laval, Quebec, Canada
[5]Infectious Disease Research Centre, Université Laval, Quebec, Canada
[*]alexandre.drouin.8@ulaval.ca

Supplementary methods

A sample compression risk bound for decision trees

Based on the pioneering work of Littlestone and Warmuth (1986)[1] and Floyd and Warmuth (1995)[2], Marchand and Sokolova (2005)[3] obtained a general sample compression bound that can be used to upper bound the generalization error (see Equation (1) of main text) of any classifier h, such that $h = \mathbf{R}(Z, \sigma)$, where $\mathbf{R}$ is a reconstruction function that unambiguously reconstructs h using a small subset Z of the training examples (referred to as the *compression set*) and a message σ of additional information. Their bound is as follows: for any data-generating distribution D, any compression set Z and message σ, we have that, with probability at least $1-\delta$ (over the random draws of S according to D^m), $R(h) \leq \varepsilon(h, S, \delta)$, with

$$\varepsilon(h,S,\delta) = 1 - \exp\left(\frac{-1}{m-|Z|-r}\left[\ln\binom{m}{|Z|} + \ln\binom{m-|Z|}{r} + \ln\left(\frac{1}{P_Z(\sigma)}\right) + \ln\left(\frac{1}{\xi(|Z|)\xi(r)\delta}\right)\right]\right), \quad \text{(S1)}$$

where $Z \subseteq S$, $P_Z(\sigma)$ is the prior probability assigned to the message σ given that the compression set is Z, $|Z|$ denotes the number of examples in the compression set Z, r is the number of prediction errors made by h on $S \setminus Z$, and

$$\xi(a) \stackrel{\text{def}}{=} \frac{6}{\pi^2}(a+1)^{-2}. \quad \text{(S2)}$$

In order to use this result to obtain a sample compression bound for k-mer-based decision tree models used in this study, we must design a message σ, and a corresponding compression set Z, that jointly allow to unambiguously reconstruct any decision tree classifier h. Recall from the main text that Z contains the genomes selected such that every k-mer in the model appears at least once in Z. Recall also that $N(Z)$ denotes the number of nucleotides contained in Z.

Our approach relies on the fact that any tree with n inner nodes admits a unique preorder enumeration of its $2n+1$ nodes (n inner nodes and $n+1$ leaves). We also consider that each message σ is given by a tuple $(n, \mathbf{v_1}, \mathbf{v_2}, \mathbf{v_3})$, where

- $\mathbf{v_1} \in \{0,1\}^{2n+1}$ is a vector that gives the type of each node in the enumeration, such that $v_{1_i} \stackrel{\text{def}}{=} 1$ if the ith node is an inner node and $v_{1_i} \stackrel{\text{def}}{=} 0$ otherwise.
- $\mathbf{v_2} \in \{1,...,c\}^{n+1}$ is a vector indicating the class predicted by each leaf in the enumeration; c is the number of classes.
- $\mathbf{v_3} \in \{1,...,N(Z)\}^n$ is a vector that specifies the k-mer used by each inner node (rule) in the enumeration, based on its position in the concatenated sequence of all genomes in Z.

Any decision tree h can then be straightforwardly reconstructed from any compression set Z and any such message tuple.

To obtain a generalization error bound, we must also define a prior probability distribution $P_Z(\sigma)$ over all possible values of σ, given a compression set Z. We start by attributing a probability of $\xi(n)$ to the number of inner nodes. Thus,

$$P_Z(\sigma) = P_Z(\sigma|n) \cdot \xi(n), \tag{S3}$$

which reflects our prior belief that smaller trees are more likely than large ones. We then assign equal probability to all messages specifying trees of n inner nodes:

$$P_Z(\sigma|n) = P_1(\mathbf{v_1}) \cdot P_2(\mathbf{v_2}) \cdot P_3(\mathbf{v_3}), \tag{S4}$$

where P_1, P_2, and P_3 are chosen as follows. First,

$$P_1(\mathbf{v_1}) = \frac{1}{\binom{2n+1}{n}}, \tag{S5}$$

which assigns equal probability to all vectors $\mathbf{v_1}$ with n elements equal to 1 and $n+1$ elements equal to 0. Then

$$P_2(\mathbf{v_2}) = \left(\frac{1}{c}\right)^{n+1}, \tag{S6}$$

which assigns equal probability to each class for the $n+1$ leaves. Finally,

$$P_3(\mathbf{v_3}) = \left(\frac{1}{N(Z)}\right)^{n}, \tag{S7}$$

which assigns equal probability over all positions in the combined sequence of all genomes in Z for every inner node. Hence, we obtain a prior $P_Z(\sigma)$, where

$$P_Z(\sigma) = \frac{6}{\pi^2}(n+1)^{-2}\binom{2n+1}{n}^{-1}\left(\frac{1}{N(Z)}\right)^{n}\left(\frac{1}{c}\right)^{n+1}. \tag{S8}$$

By inserting this prior $P_Z(\sigma)$ into Equation S1, we obtain a sample compression risk bound $\varepsilon_{CART}(h,S,\delta)$, which valid for any decision tree h based on rules that detect the presence of k-mers:

$$\varepsilon_{CART}(h,S,\delta) = 1 - \exp\left(\frac{-1}{m-|Z|-r}\left[\ln\binom{m}{|Z|} + \ln\binom{m-|Z|}{r} + \ln\binom{2n+1}{n} + n \cdot \ln(N(Z)) + (n+1)\ln(c) + \ln\left(\frac{\pi^6(n+1)^2(r+1)^2(|Z|+1)^2}{216\cdot\delta}\right)\right]\right). \tag{S9}$$

In the main text, we use $|h|$, instead of n, for the number of rules (i.e., internal nodes) in the decision tree h.

Related work on generalization bounds for decision trees

Several theoretical upper bounds on the risk of decision trees exist in the literature. Most of them are either based on the Vapnik-Chervonenkis dimension[4] (VC-dim) or the Rademacher complexity[5]. However, the tightness of these bounds is challenged in our setting due to the extremely high dimensionality of the input space. Given that our goal is to derive a generalization bound that is tight enough to guide model selection, including the pruning process of decision trees, such bounds are of limited interest. In fact, the VC-dim and Rademacher complexity are only defined for data-independent sets of classifiers. Given that we consider k-mers of length $k = 31$, we have to consider all decision trees of n nodes (for small n) that can be constructed over $d = 4^{31}$ boolean variables (i.e., the presence or absence of a k-mer). According to the recent work of Yıldız (2015)[6], the VC-dim of decision trees of height p is a least $2^{(p-1)}(1+\lfloor\log_2(d-p+2)\rfloor)$. Since p is at least $\log_2(n)$, this gives a linear increase in n with a large multiplier, since $d = 4^{31}$. Similarly, bounds based on Rademacher complexities exhibit the same difficulties[7].

One way to obtain tighter bounds for data-independent sets of classifiers is to use part of the training set to build the model and the remaining data to calculate a generalization bound. For instance, Kääriäinen et al. (2004)[8] use a fraction of the training set to build a (possibly very large) decision tree T and then use the remaining data to prune it based on the Rademacher complexity

of the set of subtrees of T. However, the problem with this approach is that fewer examples are used to grow the tree and then prune it.

Consequently, in order to use the full training set to grow and prune the tree, while achieving a tight bound, we have decided to investigate risk bounds for data-dependent sets of classifiers. Such sets of classifiers can be significantly more concise than their data-independent counterparts and thus, lead to tighter bounds. In our case, we consider the set of all decision trees composed exclusively of k-mers that are present in the data set (and not the full set of 4^{31} k-mers). In this sense, our best prospect was the sample compression bounds proposed by Floyd and Warmuth (1995)[2] and then later by Marchand and Sokolova (2005)[3] for conjunctions, disjunctions, and decision lists. Such bounds were recently explored by Drouin et al. (2016)[9] to obtain tight generalization bounds for conjunctions and disjunctions of k-mers learned using Set Covering Machines[10]. In the case of decision trees, the only sample compression bound that we are aware of is the one proposed by Shah (2007)[11]. However, this bound is too generic and no pruning algorithm based on it was ever proposed. Instead, following the work of Drouin et al. (2016)[9], we have decided to propose a tighter bound, specialized to decision trees of k-mers, that takes into account the particularities of this representation, i.e., that k-mers are substrings of genome sequences.

In fact, the main difference between our bound and the one of Shah (2007)[11] is in the way that the decision tree is encoded into a compression set and a message string. In their bound, the k-mers are considered as generic features and the message string contains the index of the k-mer on which each rule (inner node) relies. The compression set serves only to specify each rule's threshold (i.e., 0 or 1) based on the value of the feature in the corresponding example. In contrast, we exploit the fact that the k-mers are substrings of genomes and define the compression set as being the smallest set of genomes that contains all the k-mers in the model. We then use the message string to specify the index of the k-mers in the concatenated sequences of the genomes of the compression set. We also account for the fact that multiple k-mers can be found in the same genome, which leads to smaller compression sets and thus a tighter bound.

More formally, for a decision tree with n inner nodes (rules) the bound of Shah (2007)[11] contains an additive term of $n \cdot \ln(d)$, where d is the total number of k-mers (i.e., 4^k), whereas, in our bound, this term is replaced by $n \cdot \ln(N(Z))$, where $N(Z)$ is the number of nucleotides in all the genomes of the compression set. Given that $|Z| \leq n$ and that we seek simple trees with few inner nodes (i.e., small n), $N(Z)$ is of the order of a few millions and is bound to be smaller than the total number of k-mers (d) when k is sufficiently large (e.g., for $k = 31$, $d = 4^k \approx 4.6116\text{e}18$), resulting in a tighter bound.

Supplementary figures

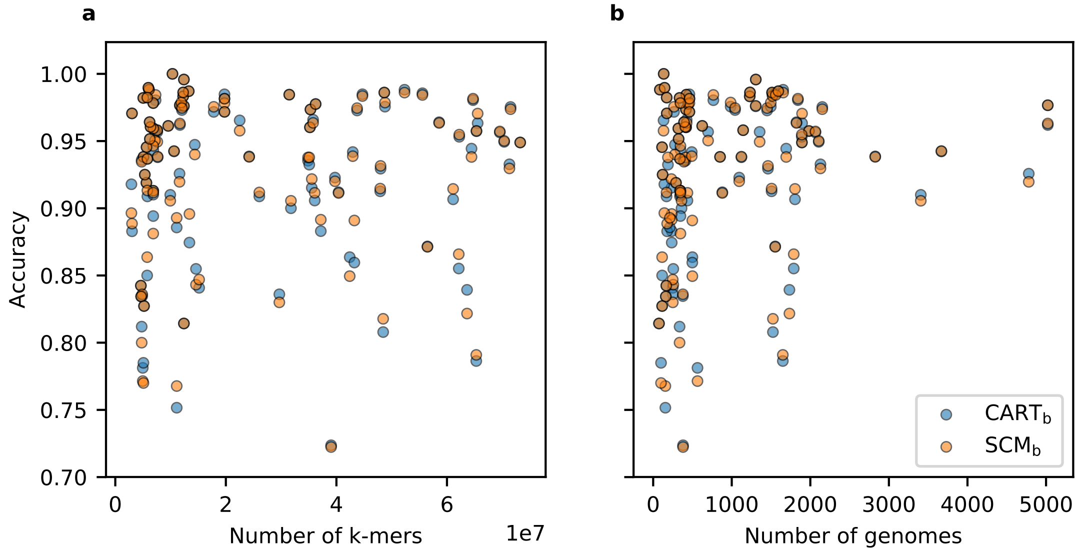

Figure S1. Accuracy of the CART$_b$ and SCM$_b$ models with respect to a) the number of *k*-mers and b) the number of genomes in each of the 107 datasets (shown as dots). Clearly, small numbers of genomes are not associated with poor accuracies. The same is true for large numbers of *k*-mers. These results emphasize the ability of these algorithms to achieve good generalization despite small samples sizes and extremely high dimensional data.

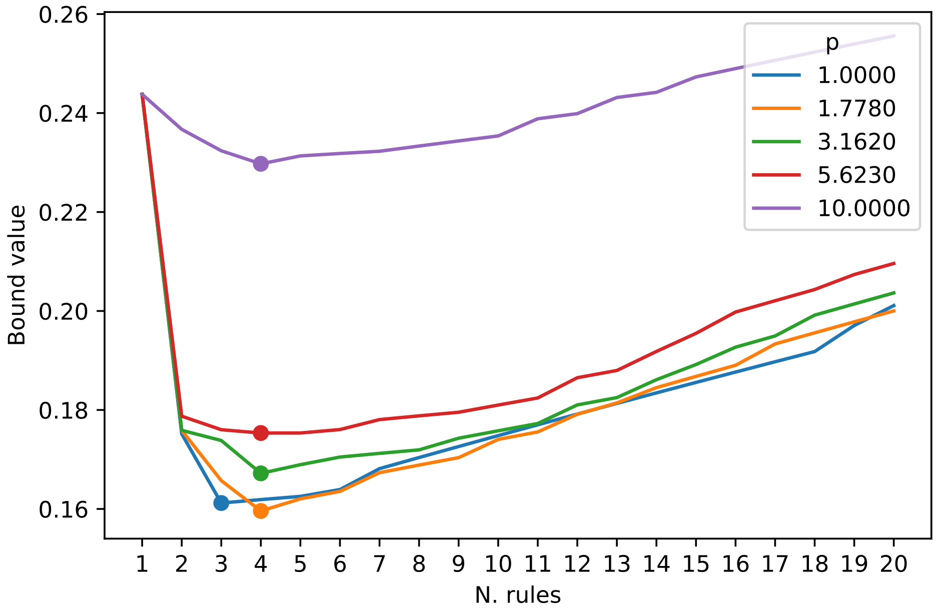

Figure S2. Value of the sample compression bound of the SCM$_b$ algorithm (Equation (2)) with respect to the number of rules in the model for the *M. tuberculosis* benchmark dataset. Each of the colored lines corresponds to a different value of the *p* hyperparameter, which controls the importance of the positive and negative classes in the greedy optimization algorithm (see Marchand and Shawe-Taylor (2002)[10]). The minimum of each line is marked by a dot. For clarity, we only show results for disjunction models (logical-OR). Clearly, there is a well-distinguishable set of hyperparameter values that yield a smaller bound value than the others and the bound allows for model selection.

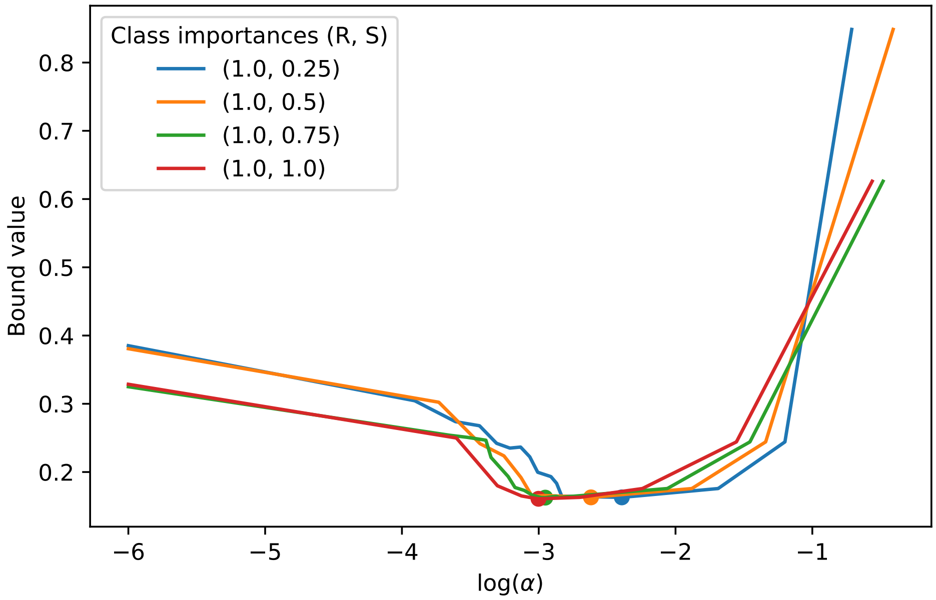

Figure S3. Value of the sample compression bound of the CART_b algorithm (Equation (3)) with respect to the alpha hyperparameter of the minimum cost-complexity pruning algorithm of Breiman et al. (1984)[12], which controls the size of the resulting tree, for the *M. tuberculosis* benchmark dataset. Each of the colored lines corresponds to a different class importance ratio, which serves to increase the importance of making errors on any of the classes. The minimum of each line is marked by a dot. For clarity, we only show results while varying the importance of the susceptible (S) class. Clearly, there is a well-distinguishable set of hyperparameter values that yield a smaller bound value than the others and the bound allows for model selection.

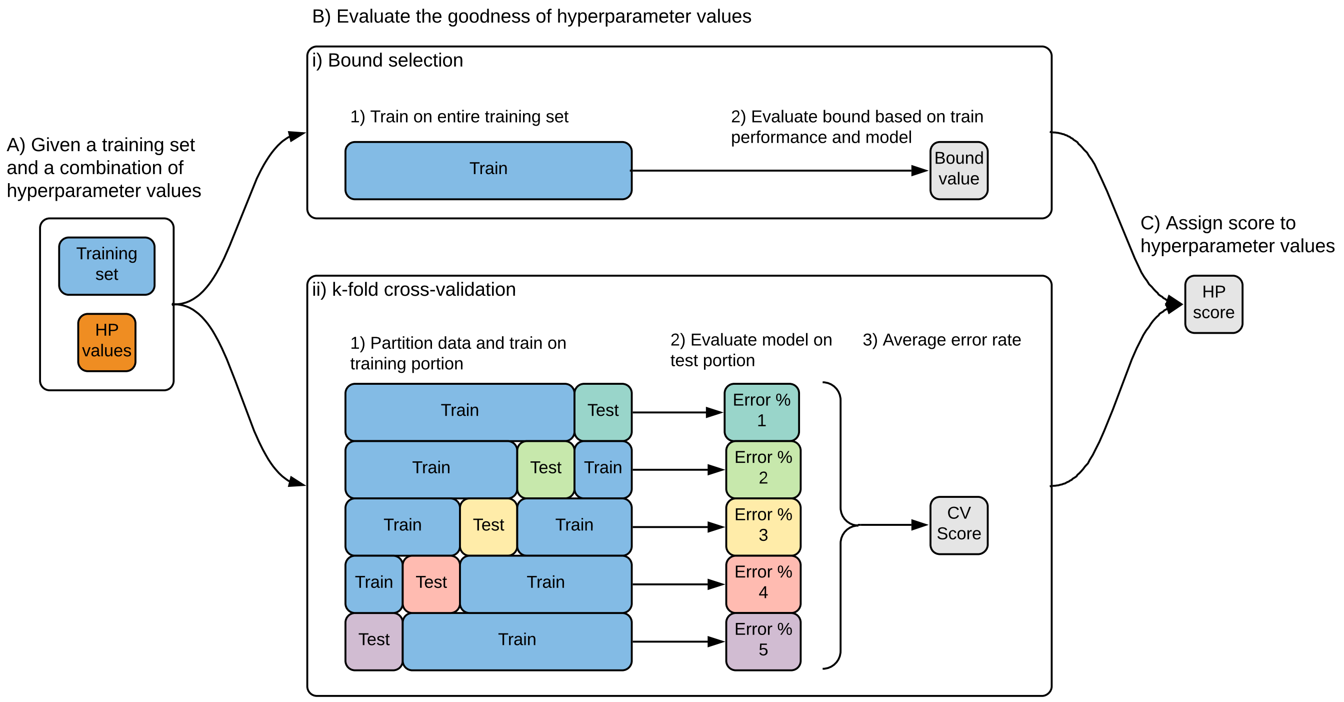

Figure S4. Illustration of the bound selection and cross-validation model selection methods. A) Both methods are given a combination of hyperparameter values to score, as well as a set of training data. B) The methods differ in the strategy that they use to compute a score. (i) Bound selection trains the algorithm on the entire training set and scores the hyperparameters based on the expression of a generalization bound. In this study, the bound depends on some properties of the model (e.g., complexity) and its performance on the training data (e.g., number of prediction errors). This requires a single training of the algorithm and all the data is used for training. (ii) In contrast, k-fold cross-validation creates k partitions of the data and trains k distinct models that are evaluated on k testing sets (folds). This approach is less computationally efficient than bound selection and requires that some data be left out for testing. C) The score estimated by both methods is assigned to the combination of hyperparameter values and the combination with the best score (e.g., minimum value) is retained.

Supplementary tables

Table S1. Detailed results for all datasets and the methods compared in Table 2 and Supplementary Table S2. For each dataset (species-antibiotic pair), the number of genomes (total, resistant, and susceptible) and *k*-mers is shown, along with the accuracy, sensitivity, specificity, F1 score, and the complexity of the models learned by each algorithm (average ± standard deviation for ten repetitions – see main text). The complexity is the number of *k*-mers used by the models, with all* indicating that feature selection was performed (see main text) and that the one million selected features were used. Missing F1 score values indicate that, in at least one repetition, the value of this metric was *nan* or infinite, which (in our case) can occur if no examples are predicted as positive or there are no true positive predictions.

Species	Antibiotic	Genomes	Resistant	Susceptible	*k*-mers (millions)	Method	Accuracy	Sensitivity	Specificity	F1 score	Complexity
A. baumannii	amikacin	256	195	61	14.6	L1-logistic	0.835±0.064	0.880±0.072	0.663±0.192	0.893±0.048	4575.7 ± 6046.6
						L2-logistic	**0.861±0.051**	0.890±0.058	**0.740±0.155**	**0.909±0.038**	all*
						Majority	0.790±0.045	**1.000±0.000**	0.000±0.000	0.882±0.029	–
						Naive Bayes	0.725±0.049	0.780±0.051	0.533±0.095	0.817±0.036	all
						Poly-SVM	**0.865±0.051**	0.902±0.056	0.717±0.173	**0.912±0.038**	all
						RBF-SVM	0.851±0.069	0.902±0.058	0.660±0.170	0.904±0.048	all
						Random Forests	0.843±0.054	0.895±0.060	0.635±0.112	0.899±0.039	6762.8 ± 7422.5
						CART$_b$	0.855±0.041	0.898±0.037	0.698±0.179	**0.907±0.027**	2.5 ± 0.5
						CART$_{cv}$	**0.867±0.041**	0.905±0.045	0.719±0.188	**0.914±0.029**	4.1 ± 1.7
						SCM$_b$	0.843±0.044	0.878±0.035	0.725±0.129	0.898±0.032	2.1 ± 0.3
						SCM$_{cv}$	0.837±0.043	0.886±0.048	0.615±0.228	0.895±0.030	5.5 ± 2.6
	ampicillin/sulbactam	155	111	44	11.1	L1-logistic	0.823±0.067	0.842±0.075	**0.756±0.190**	0.876±0.046	106216.1 ± 233553.8
						L2-logistic	0.835±0.056	0.867±0.069	0.743±0.160	0.887±0.039	all*
						Majority	0.748±0.042	**1.000±0.000**	0.000±0.000	0.855±0.028	–
						Naive Bayes	0.797±0.071	0.902±0.082	0.492±0.175	0.868±0.050	all
						Poly-SVM	0.829±0.053	0.867±0.059	0.722±0.140	0.883±0.040	all
						RBF-SVM	**0.848±0.034**	0.893±0.063	0.706±0.187	**0.898±0.024**	all
						Random Forests	**0.839±0.059**	0.881±0.081	0.706±0.135	**0.890±0.042**	1910.0 ± 3035.0
						CART$_b$	0.752±0.046	0.811±0.076	0.586±0.285	0.830±0.030	1.0 ± 0.0
						CART$_{cv}$	0.810±0.084	0.846±0.100	0.707±0.141	0.867±0.063	7.3 ± 2.9
						SCM$_b$	0.768±0.060	0.841±0.073	0.562±0.227	0.843±0.042	1.0 ± 0.0
						SCM$_{cv}$	0.787±0.049	0.842±0.067	0.624±0.218	0.855±0.031	5.6 ± 2.4
	carbapenem	232	122	110	35.5	L1-logistic	0.943±0.040	0.937±0.043	0.948±0.047	0.949±0.037	1075.4 ± 627.9
						L2-logistic	0.943±0.046	0.945±0.048	0.942±0.049	0.950±0.038	all*
						Majority	0.520±0.094	0.900±0.316	0.100±0.316	–	–
						Naive Bayes	0.904±0.026	**0.977±0.032**	0.810±0.056	0.918±0.025	all
						Poly-SVM	0.948±0.040	0.949±0.046	0.940±0.055	0.954±0.036	all
						RBF-SVM	0.946±0.039	0.945±0.050	0.946±0.042	0.950±0.035	all
						Random Forests	**0.965±0.031**	**0.968±0.038**	**0.958±0.047**	**0.969±0.029**	1637.7 ± 2268.4
						CART$_b$	0.915±0.052	0.905±0.054	0.925±0.078	0.922±0.049	2.0 ± 0.0
						CART$_{cv}$	0.915±0.054	0.918±0.061	0.910±0.070	0.923±0.049	2.2 ± 1.5
						SCM$_b$	0.922±0.047	0.914±0.042	0.929±0.078	0.929±0.041	2.0 ± 0.0
						SCM$_{cv}$	0.924±0.040	0.917±0.050	0.923±0.062	0.928±0.040	3.5 ± 1.1
	ceftazidime	277	249	28	14.4	L1-logistic	**0.944±0.034**	0.990±0.017	0.511±0.288	**0.969±0.018**	153574.8 ± 295176.4
						L2-logistic	0.927±0.049	0.968±0.047	0.548±0.310	0.960±0.029	all*
						Majority	0.907±0.035	**1.000±0.000**	0.000±0.000	0.951±0.019	–
						Naive Bayes	0.871±0.039	0.858±0.041	**1.000±0.000**	0.923±0.024	all

Continued on next page

Table S1. (Continued)

Species	Antibiotic	Genomes	Resistant	Susceptible	k-mers (millions)	Method	Accuracy	Sensitivity	Specificity	F1 score	Complexity
						Poly-SVM	**0.942±0.032**	0.982±0.029	0.562±0.284	**0.968±0.017**	all
						RBF-SVM	**0.942±0.033**	0.980±0.030	0.577±0.289	**0.968±0.018**	all
						Random Forests	0.940±0.021	0.982±0.022	0.546±0.260	**0.967±0.012**	1389.7 ± 2594.2
						CART$_{b}$	**0.947±0.026**	0.984±0.022	0.593±0.229	**0.971±0.014**	1.1 ± 0.3
						CART$_{cv}$	**0.951±0.047**	0.974±0.042	0.736±0.280	**0.973±0.027**	2.3 ± 1.3
						SCM$_{b}$	0.940±0.043	0.976±0.043	0.593±0.229	**0.967±0.024**	1.2 ± 0.4
						SCM$_{cv}$	0.935±0.042	0.967±0.042	0.660±0.277	**0.964±0.024**	1.7 ± 1.1
	imipenem	499	325	174	42.4	L1-logistic	0.880±0.029	0.915±0.031	0.819±0.059	0.907±0.025	3980.5 ± 4676.0
						L2-logistic	**0.885±0.034**	0.906±0.038	**0.851±0.070**	**0.909±0.029**	all*
						Majority	0.644±0.037	**1.000±0.000**	0.000±0.000	0.783±0.028	–
						Naive Bayes	0.822±0.027	0.912±0.031	0.661±0.057	0.868±0.023	all
						Poly-SVM	**0.886±0.031**	0.917±0.028	0.832±0.072	**0.912±0.024**	all
						RBF-SVM	0.880±0.031	0.912±0.041	0.824±0.063	0.907±0.024	all
						Random Forests	**0.892±0.024**	0.937±0.023	0.812±0.052	**0.917±0.020**	6314.6 ± 7055.6
						CART$_{b}$	0.864±0.042	0.915±0.039	0.773±0.110	0.896±0.032	3.4 ± 0.7
						CART$_{cv}$	0.863±0.041	0.910±0.035	0.780±0.085	0.894±0.033	9.6 ± 5.0
						SCM$_{b}$	0.849±0.031	0.926±0.020	0.711±0.078	0.888±0.023	2.7 ± 0.5
						SCM$_{cv}$	0.857±0.039	0.912±0.046	0.759±0.075	0.890±0.033	10.6 ± 5.2
	meropenem	236	203	33	13.4	L1-logistic	0.896±0.048	0.933±0.042	**0.628±0.208**	0.940±0.028	167820.2 ± 298168.7
						L2-logistic	0.887±0.049	0.932±0.046	0.560±0.240	0.935±0.030	all*
						Majority	0.881±0.025	**1.000±0.000**	0.000±0.000	0.936±0.014	–
						Naive Bayes	0.791±0.048	0.836±0.054	0.451±0.269	0.876±0.030	all
						Poly-SVM	0.900±0.050	0.945±0.019	0.580±0.364	0.944±0.027	all
						RBF-SVM	0.906±0.042	0.954±0.021	0.566±0.355	**0.948±0.022**	all
						Random Forests	**0.921±0.027**	0.974±0.017	0.541±0.269	**0.956±0.014**	2282.4 ± 3017.3
						CART$_{b}$	0.874±0.032	0.964±0.040	0.245±0.334	0.931±0.017	0.9 ± 0.9
						CART$_{cv}$	0.900±0.032	0.943±0.036	0.583±0.220	0.943±0.018	6.5 ± 2.9
						SCM$_{b}$	0.896±0.038	0.947±0.029	0.499±0.327	0.941±0.021	1.5 ± 0.5
						SCM$_{cv}$	0.889±0.036	0.943±0.041	0.507±0.255	0.938±0.020	4.5 ± 2.5
	tobramycin	249		46	15.2	L1-logistic	0.863±0.019	0.905±0.047	0.648±0.159	0.918±0.012	70944.3 ± 159229.9
						L2-logistic	0.857±0.049	0.882±0.063	**0.741±0.140**	0.912±0.030	all*
						Majority	0.849±0.034	**1.000±0.000**	0.000±0.000	0.918±0.020	–
						Naive Bayes	0.733±0.049	0.784±0.067	0.457±0.208	0.831±0.038	all
						Poly-SVM	**0.873±0.054**	0.921±0.055	0.606±0.226	**0.925±0.032**	all
						RBF-SVM	**0.873±0.043**	0.935±0.051	0.520±0.177	**0.926±0.027**	all
						Random Forests	**0.882±0.039**	0.945±0.039	0.539±0.183	**0.931±0.024**	1034.8 ± 901.9
						CART$_{b}$	0.841±0.041	0.936±0.050	0.342±0.307	0.909±0.024	1.5 ± 0.7
						CART$_{cv}$	0.841±0.051	0.881±0.059	0.627±0.138	0.903±0.032	6.8 ± 2.3
						SCM$_{b}$	0.847±0.040	0.924±0.042	0.441±0.272	0.911±0.023	1.7 ± 0.5
						SCM$_{cv}$	0.841±0.049	0.871±0.061	0.698±0.187	0.902±0.031	6.0 ± 2.9
E. coli	amoxicillin	1095	661	434	39.7	L1-logistic	0.900±0.029	0.874±0.028	0.942±0.054	0.914±0.026	1861.0 ± 4505.5
						L2-logistic	0.888±0.022	0.873±0.028	0.912±0.040	0.905±0.020	all*
						Majority	0.614±0.025	**1.000±0.000**	0.000±0.000	0.761±0.020	–
						Naive Bayes	0.603±0.025	0.552±0.025	0.685±0.043	0.630±0.027	all
						Poly-SVM	0.869±0.032	0.888±0.029	0.842±0.058	0.893±0.027	all

Continued on next page

Table S1. (Continued)

Species	Antibiotic	Genomes	Resistant	Susceptible	k-mers (millions)	Method	Accuracy	Sensitivity	Specificity	F1 score	Complexity
						RBF-SVM	0.864±0.039	0.878±0.034	0.844±0.054	0.888±0.034	all
						Random Forests	0.909±0.022	0.893±0.026	0.934±0.029	0.923±0.019	17109.2 ± 13709.9
						CART$_b$	**0.923±0.018**	0.891±0.026	**0.973±0.011**	**0.934±0.016**	3.6 ± 0.5
						CART$_{cv}$	**0.919±0.022**	0.889±0.024	**0.966±0.027**	**0.930±0.019**	4.1 ± 1.7
						SCM$_b$	**0.920±0.016**	0.893±0.025	0.962±0.014	**0.932±0.015**	4.1 ± 0.7
						SCM$_{cv}$	**0.920±0.021**	0.891±0.023	**0.966±0.028**	**0.932±0.019**	4.0 ± 1.2
	amoxicillin/clavulan-ic acid	1524	464	1060	48.5	L1-logistic	0.792±0.018	**0.746±0.075**	0.812±0.026	**0.683±0.040**	3727.2 ± 5890.3
						L2-logistic	0.789±0.022	0.684±0.078	0.835±0.042	0.661±0.035	all*
						Majority	0.697±0.014	0.000±0.000	**1.000±0.000**	–	–
						Naive Bayes	0.634±0.026	0.596±0.035	0.652±0.054	0.497±0.017	all
						Poly-SVM	0.779±0.022	0.604±0.070	0.856±0.020	0.622±0.043	all
						RBF-SVM	0.776±0.021	0.597±0.073	0.855±0.016	0.616±0.046	all
						Random Forests	0.812±0.021	0.598±0.060	0.906±0.023	0.657±0.037	39289.6 ± 29690.9
						CART$_b$	0.808±0.021	0.563±0.075	0.915±0.041	0.638±0.040	7.0 ± 0.7
						CART$_{cv}$	0.812±0.019	0.533±0.101	0.933±0.052	0.627±0.047	13.3 ± 7.7
						SCM$_b$	0.818±0.019	0.464±0.050	0.972±0.014	0.606±0.041	4.6 ± 1.1
						SCM$_{cv}$	**0.830±0.023**	0.467±0.059	0.988±0.010	0.623±0.054	6.2 ± 1.9
	ampicillin	436	271	165	36.1	L1-logistic	**0.926±0.029**	0.905±0.052	**0.964±0.031**	**0.937±0.027**	3006.9 ± 2011.3
						L2-logistic	0.908±0.038	0.900±0.051	0.920±0.049	0.922±0.034	all*
						Majority	0.610±0.040	**1.000±0.000**	0.000±0.000	0.757±0.031	–
						Naive Bayes	0.629±0.036	0.615±0.058	0.651±0.075	0.668±0.035	all
						Poly-SVM	0.826±0.029	0.839±0.046	0.808±0.035	0.855±0.025	all
						RBF-SVM	0.824±0.027	0.839±0.040	0.803±0.042	0.853±0.023	all
						Random Forests	0.923±0.042	0.913±0.051	0.938±0.051	**0.935±0.038**	2720.0 ± 6356.9
						CART$_b$	0.906±0.037	0.902±0.044	0.910±0.062	0.921±0.033	2.2 ± 0.6
						CART$_{cv}$	0.916±0.036	0.912±0.042	0.922±0.065	0.930±0.029	3.1 ± 1.7
						SCM$_b$	0.911±0.040	0.912±0.048	0.911±0.063	0.926±0.035	2.2 ± 0.6
						SCM$_{cv}$	**0.933±0.040**	0.933±0.043	0.936±0.065	**0.944±0.033**	3.5 ± 1.4
	aztreonam	419	39	380	35.2	L1-logistic	**0.954±0.011**	0.673±0.148	0.984±0.010	0.725±0.088	13896.1 ± 22296.9
						L2-logistic	**0.953±0.024**	0.649±0.173	0.985±0.011	0.713±0.153	all*
						Majority	0.906±0.018	0.000±0.000	**1.000±0.000**	–	–
						Naive Bayes	0.765±0.086	**0.854±0.135**	0.755±0.089	0.418±0.128	all
						Poly-SVM	0.934±0.024	0.497±0.223	0.980±0.025	0.556±0.207	all
						RBF-SVM	0.931±0.027	0.432±0.304	0.984±0.019	–	all
						Random Forests	**0.959±0.021**	0.628±0.133	**0.995±0.009**	0.745±0.113	4960.1 ± 4877.3
						CART$_b$	**0.960±0.021**	0.696±0.118	0.988±0.012	**0.768±0.105**	1.0 ± 0.0
						CART$_{cv}$	**0.958±0.023**	0.696±0.118	0.985±0.015	**0.759±0.108**	1.6 ± 1.3
						SCM$_b$	**0.960±0.021**	0.696±0.118	0.988±0.012	**0.768±0.105**	1.0 ± 0.0
						SCM$_{cv}$	**0.958±0.021**	0.710±0.110	0.984±0.012	**0.760±0.101**	1.8 ± 1.0
	cefalotin	250	59	191	29.7	L1-logistic	0.804±0.056	0.548±0.149	0.884±0.050	0.571±0.124	592.7 ± 753.2
						L2-logistic	0.812±0.074	**0.565±0.157**	0.893±0.058	0.594±0.150	all*
						Majority	0.752±0.056	0.000±0.000	**1.000±0.000**	–	–
						Naive Bayes	0.834±0.045	**0.558±0.156**	0.919±0.046	0.613±0.126	all
						Poly-SVM	**0.840±0.037**	0.485±0.114	0.955±0.025	0.589±0.118	all

Continued on next page

Table S1. (Continued)

Species	Antibiotic	Genomes	Resistant	Susceptible	k-mers (millions)	Method	Accuracy	Sensitivity	Specificity	F1 score	Complexity
						RBF-SVM	0.836±0.030	0.492±0.112	0.947±0.028	0.586±0.111	all
						Random Forests	**0.846±0.042**	0.502±0.138	0.956±0.035	0.604±0.145	1334.6 ± 2911.6
						CART$_b$	0.836±0.048	0.530±0.131	0.933±0.041	0.608±0.128	1.0 ± 0.0
						CART$_{cv}$	0.836±0.044	0.524±0.120	0.936±0.041	0.601±0.124	2.0 ± 1.5
						SCM$_b$	0.830±0.047	0.520±0.137	0.927±0.034	0.594±0.130	1.0 ± 0.0
						SCM$_{cv}$	**0.840±0.057**	0.538±0.092	0.941±0.045	**0.626±0.106**	3.2 ± 1.4
	cefepime	426	32	394	35.8	L1-logistic	**0.971±0.022**	0.825±0.163	0.981±0.021	0.795±0.133	3594.5 ± 4097.0
						L2-logistic	**0.975±0.019**	0.754±0.215	0.990±0.013	0.786±0.205	all*
						Majority	0.934±0.019	0.000±0.000	**1.000±0.000**	–	–
						Naive Bayes	0.782±0.049	0.679±0.197	0.790±0.052	0.293±0.115	all
						Poly-SVM	0.966±0.015	0.576±0.259	**0.994±0.009**	0.657±0.188	all
						RBF-SVM	0.966±0.016	0.565±0.258	**0.995±0.009**	0.657±0.186	all
						Random Forests	**0.979±0.013**	0.754±0.173	**0.995±0.011**	**0.823±0.103**	1626.6 ± 2320.9
						CART$_b$	0.966±0.016	0.728±0.144	0.982±0.017	0.736±0.083	1.4 ± 0.7
						CART$_{cv}$	**0.973±0.014**	**0.841±0.153**	0.982±0.012	0.800±0.100	3.4 ± 1.2
						SCM$_b$	0.964±0.017	0.705±0.199	0.981±0.017	0.709±0.130	1.5 ± 0.5
						SCM$_{cv}$	0.965±0.018	0.768±0.299	0.978±0.018	–	2.1 ± 0.3
	cefotaxime	1450	139	1311	43.7	L1-logistic	**0.976±0.009**	**0.860±0.088**	0.988±0.006	0.873±0.060	67531.0 ± 138221.3
						L2-logistic	**0.973±0.010**	**0.860±0.086**	0.985±0.006	0.859±0.062	all*
						Majority	0.898±0.018	0.000±0.000	**1.000±0.000**	–	–
						Naive Bayes	0.830±0.048	0.754±0.105	0.837±0.061	0.477±0.060	all
						Poly-SVM	**0.971±0.011**	0.816±0.101	0.988±0.006	0.846±0.067	all
						RBF-SVM	0.969±0.010	0.803±0.091	0.987±0.007	0.833±0.065	all
						Random Forests	**0.979±0.011**	0.850±0.089	**0.993±0.005**	**0.890±0.059**	12400.7 ± 11836.2
						CART$_b$	**0.973±0.009**	0.782±0.089	**0.994±0.004**	0.849±0.058	2.8 ± 0.9
						CART$_{cv}$	**0.979±0.008**	**0.858±0.092**	**0.993±0.003**	**0.890±0.051**	6.0 ± 2.4
						SCM$_b$	**0.974±0.007**	0.786±0.074	**0.995±0.004**	0.858±0.044	3.0 ± 1.1
						SCM$_{cv}$	**0.980±0.009**	**0.860±0.094**	**0.993±0.003**	**0.892±0.053**	5.2 ± 1.2
	cefoxitin	417	27	390	35.3	L1-logistic	0.964±0.025	0.682±0.232	0.982±0.017	0.686±0.198	1584.0 ± 3147.2
						L2-logistic	0.945±0.021	0.489±0.219	0.974±0.018	0.500±0.175	all*
						Majority	0.940±0.015	0.000±0.000	**1.000±0.000**	–	–
						Naive Bayes	0.810±0.038	0.381±0.234	0.836±0.036	–	all
						Poly-SVM	0.965±0.012	0.400±0.258	**0.999±0.004**	–	all
						RBF-SVM	0.967±0.013	0.450±0.247	**0.999±0.004**	–	all
						Random Forests	0.961±0.017	0.407±0.274	**0.996±0.009**	–	788.2 ± 1579.9
						CART$_b$	**0.973±0.018**	**0.794±0.130**	0.985±0.013	0.782±0.131	1.0 ± 0.0
						CART$_{cv}$	0.971±0.017	**0.799±0.129**	0.982±0.015	0.769±0.123	1.7 ± 0.9
						SCM$_b$	**0.973±0.018**	**0.794±0.130**	0.985±0.013	0.782±0.131	1.0 ± 0.0
						SCM$_{cv}$	**0.982±0.014**	0.744±0.182	**0.996±0.012**	**0.816±0.143**	2.0 ± 0.0
	cefuroxime	1507	241	1266	47.9	L1-logistic	0.833±0.021	0.333±0.289	0.928±0.065	–	11536.9 ± 14007.2
						L2-logistic	0.830±0.028	0.504±0.111	0.893±0.036	0.486±0.085	all*
						Majority	0.838±0.020	0.000±0.000	**1.000±0.000**	–	–
						Naive Bayes	0.793±0.022	**0.578±0.096**	0.834±0.031	0.472±0.054	all
						Poly-SVM	0.880±0.018	0.395±0.073	0.974±0.013	0.514±0.083	all
						RBF-SVM	0.875±0.011	0.392±0.062	0.968±0.012	0.500±0.059	all

Continued on next page

Table S1. (Continued)

Species	Antibiotic	Genomes	Resistant	Susceptible	k-mers (millions)	Method	Accuracy	Sensitivity	Specificity	F1 score	Complexity
						Random Forests	0.904±0.012	0.439±0.063	**0.994±0.005**	0.594±0.056	8889.5 ± 13173.8
						CART$_b$	**0.913±0.015**	0.502±0.055	**0.992±0.004**	0.650±0.053	3.0 ± 0.5
						CART$_{cv}$	**0.916±0.011**	0.534±0.058	0.990±0.006	**0.672±0.047**	5.5 ± 1.4
						SCM$_b$	**0.915±0.015**	0.515±0.052	**0.992±0.004**	0.660±0.051	3.4 ± 0.8
						SCM$_{cv}$	**0.917±0.012**	0.545±0.064	0.989±0.004	**0.679±0.052**	5.5 ± 1.2
	ceftazidime	1497	99	1398	48.8	L1-logistic	0.976±0.010	0.794±0.136	0.989±0.007	0.812±0.093	47016.4 ± 110544.7
						L2-logistic	0.974±0.008	0.743±0.126	0.990±0.008	0.786±0.087	all*
						Majority	0.932±0.010	0.000±0.000	**1.000±0.000**	–	–
						Naive Bayes	0.768±0.023	**0.905±0.114**	0.758±0.025	0.343±0.047	all
						Poly-SVM	0.966±0.008	0.636±0.097	0.990±0.009	0.710±0.080	all
						RBF-SVM	0.966±0.008	0.639±0.088	0.989±0.008	0.712±0.074	all
						Random Forests	**0.988±0.007**	0.840±0.108	**0.998±0.002**	**0.895±0.064**	7770.6 ± 8004.7
						CART$_b$	0.976±0.010	0.723±0.140	**0.994±0.004**	0.789±0.105	2.8 ± 0.4
						CART$_{cv}$	**0.983±0.005**	0.826±0.082	**0.994±0.006**	0.867±0.045	5.8 ± 1.9
						SCM$_b$	**0.979±0.010**	0.730±0.128	**0.996±0.004**	0.814±0.101	2.8 ± 0.6
						SCM$_{cv}$	**0.985±0.005**	0.830±0.065	**0.996±0.005**	0.882±0.043	3.8 ± 0.6
	ciprofloxacin	1519	289	1230	44.7	L1-logistic	**0.986±0.006**	**0.949±0.028**	**0.995±0.005**	**0.962±0.018**	432.7 ± 1043.6
						L2-logistic	0.963±0.012	0.868±0.065	0.986±0.010	0.900±0.031	all*
						Majority	0.806±0.021	0.000±0.000	**1.000±0.000**	–	–
						Naive Bayes	0.835±0.029	0.911±0.052	0.817±0.043	0.682±0.048	all
						Poly-SVM	0.965±0.014	0.845±0.061	**0.994±0.006**	0.902±0.035	all
						RBF-SVM	0.965±0.014	0.850±0.060	**0.993±0.008**	0.903±0.034	all
						Random Forests	0.975±0.011	0.892±0.051	**0.996±0.006**	0.932±0.029	6291.3 ± 11483.4
						CART$_b$	**0.985±0.005**	0.938±0.022	**0.996±0.005**	**0.960±0.013**	2.0 ± 0.0
						CART$_{cv}$	**0.983±0.005**	0.935±0.022	**0.996±0.005**	**0.957±0.012**	2.2 ± 0.4
						SCM$_b$	**0.983±0.005**	0.938±0.022	**0.995±0.005**	**0.956±0.013**	2.0 ± 0.0
						SCM$_{cv}$	**0.983±0.004**	0.939±0.023	**0.995±0.005**	**0.957±0.010**	2.7 ± 1.1
	gentamicin	1513	115	1398	48.7	L1-logistic	**0.983±0.007**	**0.896±0.053**	**0.991±0.005**	0.891±0.043	5673.3 ± 13968.4
						L2-logistic	**0.979±0.007**	0.851±0.068	**0.991±0.005**	0.864±0.045	all*
						Majority	0.923±0.011	0.000±0.000	**1.000±0.000**	–	–
						Naive Bayes	0.687±0.037	0.824±0.048	0.676±0.043	0.289±0.038	all
						Poly-SVM	0.956±0.014	0.614±0.152	0.986±0.006	0.676±0.104	all
						RBF-SVM	0.957±0.013	0.619±0.134	0.986±0.005	0.686±0.096	all
						Random Forests	**0.987±0.007**	**0.900±0.070**	**0.995±0.005**	**0.914±0.055**	4242.2 ± 5881.0
						CART$_b$	**0.986±0.006**	**0.898±0.061**	**0.994±0.004**	**0.907±0.046**	2.0 ± 0.0
						CART$_{cv}$	**0.985±0.009**	**0.893±0.064**	**0.993±0.007**	0.898±0.063	2.4 ± 1.3
						SCM$_b$	**0.986±0.006**	**0.898±0.061**	**0.994±0.004**	**0.907±0.046**	2.0 ± 0.0
						SCM$_{cv}$	**0.986±0.007**	**0.898±0.061**	**0.993±0.005**	**0.905±0.049**	2.2 ± 0.6
	meropenem	446	28	418	36.2	L1-logistic	**0.982±0.013**	**0.963±0.078**	0.983±0.015	0.844±0.093	445.5 ± 394.3
						L2-logistic	0.976±0.011	0.787±0.189	0.987±0.009	0.761±0.118	all*
						Majority	0.949±0.012	0.000±0.000	**1.000±0.000**	–	–
						Naive Bayes	0.811±0.046	0.838±0.121	0.809±0.047	0.316±0.101	all
						Poly-SVM	**0.984±0.009**	0.770±0.184	**0.996±0.006**	0.823±0.098	all
						RBF-SVM	**0.983±0.010**	0.745±0.203	**0.996±0.008**	0.806±0.105	all
						Random Forests	**0.990±0.006**	0.863±0.127	**0.996±0.006**	**0.883±0.093**	2669.0 ± 2582.8

Continued on next page

Table S1. (Continued)

Species	Antibiotic	Genomes	Resistant	Susceptible	k-mers (millions)	Method	Accuracy	Sensitivity	Specificity	F1 score	Complexity
						CART$_b$	0.978 ± 0.013	0.922 ± 0.130	0.981 ± 0.014	0.802 ± 0.097	1.4 ± 0.5
						CART$_{cv}$	0.976 ± 0.013	0.907 ± 0.158	0.981 ± 0.014	0.791 ± 0.103	1.6 ± 1.1
						SCM$_b$	0.978 ± 0.013	0.922 ± 0.130	0.981 ± 0.014	0.802 ± 0.097	1.1 ± 0.3
						SCM$_{cv}$	0.973 ± 0.012	0.840 ± 0.152	0.981 ± 0.016	0.758 ± 0.069	1.8 ± 0.8
	piperacillin/tazobactam	1461	99	1362	48.0	L1-logistic	**0.929 ± 0.012**	0.000 ± 0.000	**1.000 ± 0.000**	–	0.0 ± 0.0
						L2-logistic	0.899 ± 0.019	0.176 ± 0.101	0.953 ± 0.016	–	all*
						Majority	**0.929 ± 0.012**	0.000 ± 0.000	**1.000 ± 0.000**	–	–
						Naive Bayes	0.675 ± 0.034	**0.481 ± 0.139**	0.689 ± 0.038	**0.173 ± 0.053**	all
						Poly-SVM	**0.933 ± 0.010**	0.066 ± 0.053	**0.999 ± 0.002**	–	all
						RBF-SVM	**0.931 ± 0.010**	0.054 ± 0.045	**0.998 ± 0.003**	–	all
						Random Forests	**0.935 ± 0.010**	0.100 ± 0.060	**0.998 ± 0.004**	–	1164.6 ± 2422.5
						CART$_b$	**0.929 ± 0.010**	0.036 ± 0.049	**0.997 ± 0.004**	–	0.5 ± 0.5
						CART$_{cv}$	**0.930 ± 0.012**	0.104 ± 0.060	**0.993 ± 0.006**	–	1.4 ± 0.7
						SCM$_b$	**0.932 ± 0.012**	0.095 ± 0.047	**0.995 ± 0.006**	–	1.0 ± 0.0
						SCM$_{cv}$	**0.933 ± 0.010**	0.099 ± 0.052	**0.996 ± 0.003**	–	1.8 ± 0.4
	tobramycin	422	50	372	31.5	L1-logistic	0.974 ± 0.014	0.892 ± 0.115	0.985 ± 0.015	0.883 ± 0.062	1686.7 ± 1223.2
						L2-logistic	0.964 ± 0.018	0.824 ± 0.124	0.982 ± 0.018	0.840 ± 0.082	all*
						Majority	0.886 ± 0.025	0.000 ± 0.000	**1.000 ± 0.000**	–	–
						Naive Bayes	0.790 ± 0.074	0.802 ± 0.166	0.787 ± 0.097	0.473 ± 0.074	all
						Poly-SVM	0.915 ± 0.027	0.536 ± 0.088	0.965 ± 0.022	0.593 ± 0.100	all
						RBF-SVM	0.912 ± 0.033	0.546 ± 0.117	0.960 ± 0.029	0.589 ± 0.124	all
						Random Forests	**0.981 ± 0.018**	0.867 ± 0.135	**0.996 ± 0.006**	0.908 ± 0.095	574.1 ± 917.1
						CART$_b$	**0.985 ± 0.014**	**0.924 ± 0.106**	**0.992 ± 0.009**	**0.927 ± 0.072**	2.0 ± 0.0
						CART$_{cv}$	**0.985 ± 0.014**	**0.924 ± 0.106**	**0.992 ± 0.009**	**0.927 ± 0.072**	2.0 ± 0.0
						SCM$_b$	**0.985 ± 0.014**	**0.924 ± 0.106**	**0.992 ± 0.009**	**0.927 ± 0.072**	2.0 ± 0.0
						SCM$_{cv}$	**0.981 ± 0.014**	0.899 ± 0.115	**0.992 ± 0.009**	0.913 ± 0.070	2.0 ± 0.0
	trimethoprim	411	147	264	34.9	L1-logistic	**0.933 ± 0.025**	**0.904 ± 0.060**	0.949 ± 0.033	**0.900 ± 0.036**	14801.2 ± 14384.9
						L2-logistic	0.911 ± 0.026	0.890 ± 0.059	0.923 ± 0.037	0.870 ± 0.039	all*
						Majority	0.662 ± 0.038	0.000 ± 0.000	**1.000 ± 0.000**	–	–
						Naive Bayes	0.711 ± 0.038	0.730 ± 0.085	0.700 ± 0.050	0.627 ± 0.064	all
						Poly-SVM	0.839 ± 0.044	0.753 ± 0.120	0.885 ± 0.049	0.755 ± 0.075	all
						RBF-SVM	0.832 ± 0.050	0.746 ± 0.124	0.877 ± 0.042	0.745 ± 0.081	all
						Random Forests	**0.929 ± 0.021**	**0.898 ± 0.057**	0.946 ± 0.027	0.893 ± 0.037	7838.8 ± 8352.5
						CART$_b$	**0.935 ± 0.021**	0.880 ± 0.058	0.963 ± 0.031	**0.901 ± 0.033**	2.0 ± 0.0
						CART$_{cv}$	0.926 ± 0.032	0.870 ± 0.072	0.955 ± 0.052	0.887 ± 0.046	2.8 ± 0.9
						SCM$_b$	**0.938 ± 0.022**	0.887 ± 0.061	0.963 ± 0.031	**0.905 ± 0.035**	2.0 ± 0.0
						SCM$_{cv}$	**0.935 ± 0.024**	0.887 ± 0.054	0.960 ± 0.033	**0.901 ± 0.039**	2.8 ± 1.4
E. faecium	vancomycin	134	51	83	10.3	L1-logistic	**1.000 ± 0.000**	**1.000 ± 0.000**	**1.000 ± 0.000**	**1.000 ± 0.000**	142.0 ± 45.2
						L2-logistic	**1.000 ± 0.000**	**1.000 ± 0.000**	**1.000 ± 0.000**	**1.000 ± 0.000**	all*
						Majority	0.588 ± 0.112	0.000 ± 0.000	**1.000 ± 0.000**	–	–
						Naive Bayes	0.808 ± 0.110	0.589 ± 0.189	0.976 ± 0.043	0.707 ± 0.159	all
						Poly-SVM	**0.996 ± 0.012**	**0.992 ± 0.024**	**1.000 ± 0.000**	**0.996 ± 0.013**	all
						RBF-SVM	**0.992 ± 0.016**	0.980 ± 0.044	**1.000 ± 0.000**	0.989 ± 0.023	all
						Random Forests	**1.000 ± 0.000**	**1.000 ± 0.000**	**1.000 ± 0.000**	**1.000 ± 0.000**	202.6 ± 491.7

Continued on next page

Table S1. (Continued)

Species	Antibiotic	Genomes	Resistant	Susceptible	*k*-mers (millions)	Method	Accuracy	Sensitivity	Specificity	F1 score	Complexity
						CART$_b$	**1.000±0.000**	**1.000±0.000**	**1.000±0.000**	**1.000±0.000**	1.0 ± 0.0
						CART$_{cv}$	**1.000±0.000**	**1.000±0.000**	**1.000±0.000**	**1.000±0.000**	1.0 ± 0.0
						SCM$_b$	**1.000±0.000**	**1.000±0.000**	**1.000±0.000**	**1.000±0.000**	1.0 ± 0.0
						SCM$_{cv}$	**1.000±0.000**	**1.000±0.000**	**1.000±0.000**	**1.000±0.000**	1.0 ± 0.0
K. pneumoniae	amikacin	1893	180	1713	73.2	L1-logistic	**0.951±0.010**	0.740±0.060	0.974±0.008	0.744±0.038	37210.7 ± 42390.4
						L2-logistic	0.942±0.010	0.694±0.068	0.968±0.010	0.693±0.051	all*
						Majority	0.904±0.014	0.000±0.000	**1.000±0.000**	–	–
						Naive Bayes	0.875±0.020	**0.910±0.046**	0.872±0.020	0.583±0.057	all
						Poly-SVM	**0.958±0.010**	0.762±0.086	0.980±0.006	**0.776±0.050**	all
						RBF-SVM	**0.957±0.010**	0.764±0.075	0.978±0.005	**0.773±0.042**	all
						Random Forests	**0.954±0.013**	0.706±0.076	0.981±0.008	0.750±0.055	10355.2 ± 16634.8
						CART$_b$	**0.949±0.011**	0.673±0.086	0.978±0.007	0.715±0.061	5.2 ± 1.5
						CART$_{cv}$	**0.951±0.015**	0.699±0.127	0.977±0.009	0.726±0.096	11.1 ± 4.0
						SCM$_b$	**0.949±0.015**	0.643±0.140	0.981±0.005	0.698±0.120	4.2 ± 0.4
						SCM$_{cv}$	**0.952±0.011**	0.642±0.062	0.985±0.008	0.720±0.058	11.3 ± 5.2
	amoxicillin/clavulan-ic acid	236	120	116	37.2	L1-logistic	**0.921±0.020**	0.937±0.042	**0.904±0.059**	**0.926±0.021**	69783.1 ± 171583.6
						L2-logistic	**0.926±0.037**	**0.953±0.043**	**0.898±0.061**	**0.930±0.038**	all*
						Majority	0.457±0.067	0.600±0.516	0.400±0.516	–	–
						Naive Bayes	0.653±0.100	0.932±0.045	0.343±0.174	0.740±0.080	all
						Poly-SVM	0.885±0.040	0.884±0.068	0.894±0.054	0.890±0.042	all
						RBF-SVM	0.891±0.052	0.888±0.071	**0.901±0.053**	0.896±0.048	all
						Random Forests	0.911±0.026	0.937±0.052	0.880±0.065	0.916±0.028	2255.7 ± 3172.6
						CART$_b$	0.883±0.046	0.904±0.084	0.855±0.101	0.890±0.050	1.8 ± 0.6
						CART$_{cv}$	0.872±0.040	0.883±0.074	0.863±0.082	0.879±0.043	2.2 ± 1.1
						SCM$_b$	0.891±0.039	0.929±0.069	0.850±0.097	0.902±0.029	1.9 ± 0.6
						SCM$_{cv}$	0.872±0.043	0.908±0.075	0.833±0.101	0.883±0.038	2.2 ± 0.9
	ampicillin/sulbactam	1654	1563	91	52.3	L1-logistic	**0.982±0.006**	0.989±0.005	0.843±0.116	**0.990±0.003**	2168.7 ± 787.6
						L2-logistic	0.970±0.010	0.984±0.006	0.709±0.202	0.984±0.005	all*
						Majority	0.952±0.011	**1.000±0.000**	0.000±0.000	0.975±0.006	–
						Naive Bayes	0.802±0.019	0.794±0.021	**0.955±0.056**	0.884±0.012	all
						Poly-SVM	0.974±0.009	0.989±0.005	0.695±0.128	**0.986±0.005**	all
						RBF-SVM	0.975±0.008	0.988±0.005	0.706±0.117	**0.987±0.004**	all
						Random Forests	**0.984±0.009**	**0.996±0.005**	0.748±0.159	**0.991±0.005**	3295.4 ± 5665.1
						CART$_b$	**0.988±0.008**	**0.997±0.004**	0.810±0.117	**0.994±0.004**	3.0 ± 0.0
						CART$_{cv}$	**0.983±0.006**	**0.991±0.007**	0.824±0.109	**0.991±0.003**	4.5 ± 3.3
						SCM$_b$	**0.986±0.008**	**0.994±0.006**	0.829±0.110	**0.993±0.004**	3.0 ± 0.5
						SCM$_{cv}$	**0.985±0.008**	**0.993±0.007**	0.829±0.110	**0.992±0.004**	3.3 ± 0.8
	aztreonam	1805	1582	223	61.1	L1-logistic	0.853±0.021	0.908±0.015	0.464±0.086	0.915±0.013	12926.6 ± 2079.5
						L2-logistic	0.884±0.014	0.953±0.016	0.394±0.093	0.935±0.009	all*
						Majority	0.876±0.020	**1.000±0.000**	0.000±0.000	0.934±0.011	–
						Naive Bayes	0.693±0.029	0.692±0.028	**0.703±0.083**	0.798±0.020	all
						Poly-SVM	0.904±0.016	0.971±0.012	0.431±0.065	**0.947±0.009**	all
						RBF-SVM	**0.908±0.014**	0.973±0.010	0.446±0.050	**0.949±0.009**	all
						Random Forests	**0.916±0.013**	0.982±0.008	0.452±0.060	**0.953±0.008**	28793.8 ± 20797.3

Continued on next page

Table S1. (Continued)

Species	Antibiotic	Genomes	Resistant	Susceptible	k-mers (millions)	Method	Accuracy	Sensitivity	Specificity	F1 score	Complexity
						CART$_b$	**0.907±0.016**	0.977±0.016	0.407±0.090	**0.948±0.010**	5.0 ± 0.7
						CART$_{cv}$	0.901±0.013	0.966±0.014	0.434±0.106	**0.944±0.008**	7.4 ± 3.3
						SCM$_b$	**0.914±0.013**	0.971±0.007	0.510±0.074	**0.952±0.008**	6.4 ± 1.4
						SCM$_{cv}$	**0.911±0.011**	0.980±0.006	0.418±0.082	**0.951±0.007**	12.4 ± 3.2
	cefazolin	1895	1706	189	65.6	L1-logistic	0.941±0.014	0.967±0.012	0.705±0.069	0.967±0.008	7038.1 ± 5605.9
						L2-logistic	0.939±0.016	0.970±0.013	0.667±0.097	0.966±0.009	all*
						Majority	0.901±0.014	**1.000±0.000**	0.000±0.000	0.948±0.008	–
						Naive Bayes	0.890±0.026	0.908±0.034	0.727±0.104	0.937±0.017	all
						Poly-SVM	**0.967±0.010**	0.979±0.008	**0.852±0.043**	**0.982±0.006**	all
						RBF-SVM	**0.967±0.010**	0.980±0.009	**0.852±0.038**	**0.982±0.006**	all
						Random Forests	0.944±0.014	0.975±0.010	0.657±0.091	0.969±0.008	22077.8 ± 15353.5
						CART$_b$	**0.963±0.010**	0.977±0.008	**0.846±0.071**	**0.980±0.006**	5.1 ± 0.6
						CART$_{cv}$	0.960±0.010	0.975±0.008	0.831±0.098	**0.978±0.005**	8.1 ± 3.4
						SCM$_b$	**0.970±0.006**	0.983±0.008	**0.853±0.061**	**0.984±0.004**	6.5 ± 1.0
						SCM$_{cv}$	**0.969±0.005**	0.983±0.008	0.835±0.060	**0.983±0.003**	7.1 ± 1.4
	cefepime	1650	1098	552	65.3	L1-logistic	0.766±0.017	0.778±0.025	0.742±0.047	0.817±0.015	5579.4 ± 9607.8
						L2-logistic	0.776±0.023	0.785±0.032	**0.758±0.035**	0.825±0.017	all*
						Majority	0.672±0.023	**1.000±0.000**	0.000±0.000	0.804±0.017	–
						Naive Bayes	0.682±0.031	0.653±0.069	0.747±0.070	0.732±0.035	all
						Poly-SVM	0.788±0.021	0.874±0.050	0.618±0.102	0.847±0.017	all
						RBF-SVM	**0.797±0.026**	0.876±0.030	0.642±0.085	**0.853±0.019**	all
						Random Forests	**0.806±0.017**	0.878±0.020	0.659±0.049	**0.859±0.012**	35037.5 ± 30125.9
						CART$_b$	0.786±0.017	0.899±0.046	0.561±0.064	0.849±0.014	6.6 ± 1.8
						CART$_{cv}$	0.788±0.020	0.897±0.056	0.569±0.095	0.850±0.017	9.4 ± 3.8
						SCM$_b$	0.791±0.023	0.942±0.026	0.483±0.044	**0.858±0.018**	3.0 ± 0.5
						SCM$_{cv}$	0.795±0.020	0.952±0.020	0.475±0.040	**0.862±0.015**	4.7 ± 1.7
	cefoxitin	1789	964	825	62.1	L1-logistic	0.857±0.017	0.825±0.044	0.897±0.050	0.864±0.019	9038.3 ± 10488.3
						L2-logistic	0.858±0.025	0.840±0.030	0.880±0.025	0.867±0.026	all*
						Majority	0.552±0.016	**1.000±0.000**	0.000±0.000	0.711±0.014	–
						Naive Bayes	0.767±0.026	0.810±0.034	0.714±0.042	0.793±0.024	all
						Poly-SVM	**0.866±0.018**	0.835±0.032	0.903±0.023	**0.872±0.020**	all
						RBF-SVM	**0.871±0.018**	0.833±0.031	0.917±0.026	**0.877±0.019**	all
						Random Forests	**0.869±0.020**	0.829±0.040	0.918±0.018	**0.874±0.022**	47666.8 ± 49259.7
						CART$_b$	0.855±0.018	0.778±0.030	0.949±0.030	0.855±0.021	5.2 ± 1.5
						CART$_{cv}$	**0.869±0.014**	0.820±0.026	0.929±0.025	**0.873±0.015**	20.6 ± 5.7
						SCM$_b$	**0.866±0.020**	0.789±0.029	**0.961±0.017**	0.866±0.022	6.5 ± 1.2
						SCM$_{cv}$	**0.872±0.017**	0.819±0.031	0.937±0.020	**0.876±0.020**	13.5 ± 3.9
	cefuroxime/sodium	1560	1469	91	55.6	L1-logistic	**0.984±0.009**	**0.991±0.006**	0.861±0.075	**0.992±0.005**	2833.1 ± 2999.7
						L2-logistic	**0.979±0.008**	0.989±0.007	0.804±0.075	**0.989±0.004**	all*
						Majority	0.948±0.011	**1.000±0.000**	0.000±0.000	0.973±0.006	–
						Naive Bayes	0.777±0.021	0.766±0.022	**0.974±0.035**	0.867±0.015	all
						Poly-SVM	0.970±0.007	0.986±0.005	0.653±0.114	**0.984±0.004**	all
						RBF-SVM	0.971±0.006	0.987±0.006	0.662±0.097	**0.985±0.004**	all
						Random Forests	**0.983±0.006**	**0.994±0.006**	0.780±0.069	**0.991±0.003**	1828.9 ± 3685.9
						CART$_b$	**0.986±0.006**	**0.996±0.003**	0.788±0.080	**0.992±0.003**	1.0 ± 0.0

Continued on next page

Table S1. (Continued)

Species	Antibiotic	Genomes	Resistant	Susceptible	k-mers (millions)	Method	Accuracy	Sensitivity	Specificity	F1 score	Complexity
						$CART_{cv}$	**0.986 ± 0.006**	**0.996 ± 0.003**	0.788 ± 0.080	**0.992 ± 0.003**	1.0 ± 0.0
						SCM_b	**0.984 ± 0.006**	**0.995 ± 0.005**	0.788 ± 0.080	**0.992 ± 0.003**	1.2 ± 0.4
						SCM_{cv}	**0.984 ± 0.007**	**0.995 ± 0.006**	0.788 ± 0.080	**0.992 ± 0.004**	2.1 ± 2.2
	ceftazidime	1983	1835	148	65.3	L1-logistic	0.956 ± 0.010	0.976 ± 0.005	0.690 ± 0.073	**0.976 ± 0.006**	7469.9 ± 4105.8
						L2-logistic	0.956 ± 0.011	0.979 ± 0.007	0.673 ± 0.126	**0.977 ± 0.006**	all*
						Majority	0.930 ± 0.014	**1.000 ± 0.000**	0.000 ± 0.000	0.963 ± 0.007	–
						Naive Bayes	0.776 ± 0.025	0.765 ± 0.025	**0.916 ± 0.060**	0.863 ± 0.016	all
						Poly-SVM	**0.966 ± 0.009**	0.988 ± 0.005	0.675 ± 0.114	**0.982 ± 0.005**	all
						RBF-SVM	**0.966 ± 0.009**	0.987 ± 0.005	0.683 ± 0.108	**0.982 ± 0.005**	all
						Random Forests	**0.965 ± 0.009**	**0.993 ± 0.003**	0.595 ± 0.105	**0.981 ± 0.005**	23866.7 ± 16647.8
						$CART_b$	**0.957 ± 0.008**	0.988 ± 0.007	0.549 ± 0.129	**0.977 ± 0.004**	4.2 ± 1.1
						$CART_{cv}$	0.951 ± 0.006	0.980 ± 0.013	0.554 ± 0.178	**0.974 ± 0.004**	7.4 ± 2.6
						SCM_b	**0.958 ± 0.008**	0.989 ± 0.008	0.549 ± 0.146	**0.977 ± 0.004**	4.8 ± 2.1
						SCM_{cv}	**0.959 ± 0.014**	0.984 ± 0.009	0.636 ± 0.138	**0.978 ± 0.008**	8.2 ± 3.3
	ceftriaxone	1842	1670	172	64.7	L1-logistic	0.972 ± 0.013	0.982 ± 0.009	0.878 ± 0.079	**0.984 ± 0.007**	3401.8 ± 2110.9
						L2-logistic	0.969 ± 0.011	0.982 ± 0.008	0.838 ± 0.068	**0.983 ± 0.006**	all*
						Majority	0.910 ± 0.016	**1.000 ± 0.000**	0.000 ± 0.000	0.953 ± 0.009	–
						Naive Bayes	0.927 ± 0.005	0.941 ± 0.012	0.789 ± 0.115	0.959 ± 0.003	all
						Poly-SVM	**0.978 ± 0.007**	0.985 ± 0.007	**0.900 ± 0.033**	**0.988 ± 0.004**	all
						RBF-SVM	**0.976 ± 0.008**	0.985 ± 0.007	0.886 ± 0.057	**0.987 ± 0.005**	all
						Random Forests	**0.975 ± 0.009**	0.986 ± 0.006	0.864 ± 0.061	**0.986 ± 0.005**	7079.7 ± 9997.1
						$CART_b$	**0.980 ± 0.007**	0.990 ± 0.006	0.881 ± 0.041	**0.989 ± 0.004**	4.9 ± 0.7
						$CART_{cv}$	**0.978 ± 0.006**	0.988 ± 0.007	0.875 ± 0.042	**0.988 ± 0.003**	8.2 ± 2.0
						SCM_b	**0.982 ± 0.007**	**0.993 ± 0.007**	0.870 ± 0.049	**0.990 ± 0.004**	5.3 ± 0.5
						SCM_{cv}	**0.981 ± 0.006**	**0.992 ± 0.008**	0.872 ± 0.043	**0.989 ± 0.003**	6.3 ± 0.8
	ciprofloxacin	2152	1817	335	71.5	L1-logistic	0.952 ± 0.013	0.972 ± 0.010	0.846 ± 0.038	0.972 ± 0.008	6018.8 ± 1859.5
						L2-logistic	0.951 ± 0.012	0.965 ± 0.011	0.873 ± 0.054	0.971 ± 0.008	all*
						Majority	0.846 ± 0.021	**1.000 ± 0.000**	0.000 ± 0.000	0.916 ± 0.012	–
						Naive Bayes	0.884 ± 0.009	0.872 ± 0.010	**0.949 ± 0.025**	0.927 ± 0.006	all
						Poly-SVM	0.960 ± 0.010	0.975 ± 0.008	0.879 ± 0.036	**0.977 ± 0.006**	all
						RBF-SVM	0.960 ± 0.011	0.976 ± 0.008	0.875 ± 0.046	**0.976 ± 0.006**	all
						Random Forests	0.957 ± 0.010	0.976 ± 0.009	0.854 ± 0.046	0.975 ± 0.006	8979.5 ± 12331.8
						$CART_b$	**0.975 ± 0.009**	0.988 ± 0.006	0.906 ± 0.046	**0.985 ± 0.005**	5.0 ± 0.9
						$CART_{cv}$	**0.972 ± 0.010**	0.983 ± 0.008	0.913 ± 0.046	**0.983 ± 0.006**	4.9 ± 1.7
						SCM_b	**0.974 ± 0.008**	0.984 ± 0.005	0.914 ± 0.038	**0.984 ± 0.005**	3.2 ± 0.4
						SCM_{cv}	**0.974 ± 0.007**	0.985 ± 0.005	0.916 ± 0.037	**0.985 ± 0.004**	3.6 ± 0.8
	ertapenem	361	288	73	31.8	L1-logistic	**0.965 ± 0.022**	0.969 ± 0.031	**0.959 ± 0.054**	**0.978 ± 0.014**	1748.0 ± 2154.3
						L2-logistic	0.939 ± 0.013	0.946 ± 0.018	0.923 ± 0.081	0.960 ± 0.009	all*
						Majority	0.783 ± 0.032	**1.000 ± 0.000**	0.000 ± 0.000	0.878 ± 0.020	–
						Naive Bayes	0.831 ± 0.041	0.825 ± 0.034	0.851 ± 0.106	0.884 ± 0.028	all
						Poly-SVM	**0.956 ± 0.016**	0.967 ± 0.019	0.919 ± 0.071	**0.972 ± 0.010**	all
						RBF-SVM	**0.957 ± 0.015**	0.967 ± 0.017	0.925 ± 0.052	**0.972 ± 0.010**	all
						Random Forests	0.940 ± 0.020	0.965 ± 0.022	0.862 ± 0.099	0.962 ± 0.013	6013.6 ± 4539.2
						$CART_b$	0.900 ± 0.019	0.937 ± 0.031	0.777 ± 0.107	0.936 ± 0.013	2.7 ± 0.8
						$CART_{cv}$	0.924 ± 0.028	0.958 ± 0.018	0.807 ± 0.130	0.952 ± 0.017	6.2 ± 1.2

Continued on next page

Table S1. (Continued)

Species	Antibiotic	Genomes	Resistant	Susceptible	k-mers (millions)	Method	Accuracy	Sensitivity	Specificity	F1 score	Complexity
						SCM$_b$	0.906±0.022	0.958±0.031	0.721±0.072	0.941±0.014	2.8 ± 0.6
						SCM$_{cv}$	0.904±0.031	0.951±0.044	0.740±0.084	0.939±0.021	4.5 ± 1.3
	gentamicin	2107	906	1201	70.3	L1-logistic	**0.952±0.010**	**0.926±0.019**	0.971±0.017	**0.943±0.011**	7607.4 ± 7145.7
						L2-logistic	**0.948±0.008**	**0.933±0.015**	0.960±0.011	**0.939±0.008**	all*
						Majority	0.571±0.015	0.000±0.000	**1.000±0.000**	–	–
						Naive Bayes	0.760±0.020	0.783±0.027	0.743±0.028	0.737±0.018	all
						Poly-SVM	0.943±0.006	0.922±0.011	0.959±0.012	0.933±0.008	all
						RBF-SVM	0.943±0.005	**0.925±0.013**	0.957±0.011	0.933±0.007	all
						Random Forests	**0.956±0.007**	**0.932±0.020**	0.974±0.010	**0.948±0.008**	42856.8 ± 31470.4
						CART$_b$	**0.949±0.007**	0.920±0.025	0.972±0.011	**0.940±0.009**	4.3 ± 1.2
						CART$_{cv}$	**0.948±0.008**	**0.931±0.025**	0.961±0.014	**0.939±0.009**	8.8 ± 3.6
						SCM$_b$	**0.950±0.007**	**0.924±0.022**	0.970±0.012	**0.941±0.009**	3.9 ± 0.7
						SCM$_{cv}$	**0.953±0.009**	**0.931±0.021**	0.970±0.017	**0.945±0.011**	7.9 ± 2.7
	imipenem	1891	660	1231	62.2	L1-logistic	**0.949±0.009**	0.920±0.018	0.964±0.012	0.927±0.012	4562.0 ± 8919.4
						L2-logistic	0.943±0.011	0.926±0.021	0.953±0.010	0.920±0.015	all*
						Majority	0.647±0.023	0.000±0.000	**1.000±0.000**	–	–
						Naive Bayes	0.771±0.021	0.611±0.034	0.858±0.019	0.652±0.035	all
						Poly-SVM	**0.951±0.008**	0.925±0.015	0.964±0.010	**0.930±0.012**	all
						RBF-SVM	**0.951±0.008**	0.927±0.015	0.964±0.010	**0.930±0.013**	all
						Random Forests	**0.949±0.008**	0.923±0.018	0.964±0.011	**0.928±0.013**	38326.0 ± 22322.1
						CART$_b$	**0.953±0.008**	**0.933±0.019**	0.964±0.009	**0.934±0.010**	2.3 ± 0.5
						CART$_{cv}$	**0.954±0.009**	**0.934±0.020**	0.966±0.010	**0.935±0.012**	3.0 ± 1.1
						SCM$_b$	**0.955±0.009**	**0.937±0.014**	0.964±0.010	**0.936±0.011**	2.0 ± 0.0
						SCM$_{cv}$	**0.956±0.009**	**0.939±0.018**	0.964±0.010	**0.937±0.011**	2.6 ± 0.8
	levofloxacin	1824	1462	362	58.6	L1-logistic	**0.964±0.007**	0.962±0.006	0.974±0.029	**0.978±0.004**	969.6 ± 1700.7
						L2-logistic	0.955±0.010	0.959±0.015	0.939±0.038	**0.972±0.007**	all*
						Majority	0.807±0.023	**1.000±0.000**	0.000±0.000	0.893±0.014	–
						Naive Bayes	0.843±0.029	0.808±0.036	**0.990±0.012**	0.892±0.023	all
						Poly-SVM	**0.960±0.011**	0.967±0.011	0.930±0.035	**0.975±0.007**	all
						RBF-SVM	**0.961±0.011**	0.967±0.013	0.931±0.024	**0.975±0.007**	all
						Random Forests	**0.960±0.011**	0.965±0.011	0.937±0.042	**0.975±0.007**	15165.3 ± 19326.5
						CART$_b$	**0.964±0.006**	0.977±0.008	0.908±0.026	**0.978±0.004**	3.1 ± 1.0
						CART$_{cv}$	**0.965±0.007**	0.977±0.008	0.915±0.037	**0.978±0.004**	3.1 ± 1.1
						SCM$_b$	**0.963±0.006**	0.976±0.009	0.911±0.031	**0.977±0.004**	2.1 ± 0.3
						SCM$_{cv}$	**0.967±0.007**	0.969±0.009	0.957±0.019	**0.979±0.004**	3.0 ± 2.9
	meropenem	2065	684	1381	69.6	L1-logistic	**0.953±0.010**	**0.924±0.037**	0.967±0.006	**0.928±0.019**	968.5 ± 67.9
						L2-logistic	**0.949±0.008**	**0.918±0.030**	0.964±0.010	0.922±0.015	all*
						Majority	0.671±0.017	0.000±0.000	**1.000±0.000**	–	–
						Naive Bayes	0.813±0.036	0.704±0.091	0.866±0.020	0.710±0.070	all
						Poly-SVM	**0.953±0.011**	0.908±0.030	0.975±0.009	**0.926±0.020**	all
						RBF-SVM	**0.951±0.012**	0.908±0.031	0.972±0.009	0.923±0.022	all
						Random Forests	**0.953±0.010**	0.912±0.031	0.973±0.007	**0.927±0.018**	20415.8 ± 24643.6
						CART$_b$	**0.956±0.011**	0.912±0.030	0.978±0.005	**0.932±0.018**	3.0 ± 0.0
						CART$_{cv}$	**0.957±0.010**	0.914±0.031	0.977±0.006	**0.932±0.019**	2.9 ± 0.6
						SCM$_b$	**0.957±0.008**	**0.924±0.026**	0.973±0.006	**0.934±0.015**	2.0 ± 0.0

Continued on next page

Table S1. (Continued)

Species	Antibiotic	Genomes	Resistant	Susceptible	k-mers (millions)	Method	Accuracy	Sensitivity	Specificity	F1 score	Complexity
						SCM_{cv}	**0.957±0.009**	**0.925±0.026**	0.973±0.006	**0.934±0.016**	2.1 ± 0.3
	nitrofurantoin	880	790	90	40.4	L1-logistic	0.894±0.010	0.940±0.015	0.491±0.148	0.940±0.006	152112.5 ± 285018.2
						L2-logistic	0.907±0.010	0.957±0.013	0.482±0.100	0.949±0.005	all*
						Majority	0.894±0.015	**1.000±0.000**	0.000±0.000	0.944±0.008	–
						Naive Bayes	0.911±0.013	0.969±0.019	0.416±0.156	0.951±0.008	all
						Poly-SVM	**0.926±0.026**	0.975±0.015	0.512±0.136	**0.959±0.015**	all
						RBF-SVM	**0.929±0.028**	0.976±0.023	**0.525±0.144**	**0.961±0.016**	all
						Random Forests	**0.923±0.019**	0.982±0.010	0.412±0.141	**0.958±0.010**	2203.2 ± 3773.1
						$CART_b$	0.912±0.025	0.965±0.018	0.457±0.116	0.951±0.015	2.8 ± 0.8
						$CART_{cv}$	0.914±0.022	0.974±0.014	0.405±0.179	**0.953±0.012**	2.8 ± 1.5
						SCM_b	0.911±0.018	0.972±0.009	0.393±0.128	0.951±0.010	2.3 ± 0.5
						SCM_{cv}	0.911±0.017	0.970±0.018	0.404±0.135	0.951±0.010	5.5 ± 4.3
	ofloxacin	74	47	27	12.4	L1-logistic	0.821±0.069	0.856±0.087	0.803±0.178	0.840±0.081	46375.4 ± 143625.1
						L2-logistic	0.793±0.109	0.792±0.176	0.811±0.199	0.801±0.133	all*
						Majority	0.600±0.155	**1.000±0.000**	0.000±0.000	0.739±0.123	–
						Naive Bayes	0.671±0.136	0.641±0.201	0.778±0.186	0.685±0.149	all
						Poly-SVM	0.807±0.126	0.847±0.116	0.764±0.208	0.827±0.119	all
						RBF-SVM	**0.850±0.119**	0.884±0.104	**0.825±0.185**	**0.870±0.101**	all
						Random Forests	0.829±0.102	0.867±0.113	0.789±0.207	0.853±0.105	192.3 ± 192.2
						$CART_b$	0.814±0.090	0.878±0.107	0.760±0.202	0.836±0.096	1.4 ± 0.5
						$CART_{cv}$	0.786±0.075	0.836±0.114	0.740±0.171	0.814±0.075	2.0 ± 0.8
						SCM_b	0.814±0.090	0.878±0.107	0.760±0.202	0.836±0.096	1.4 ± 0.5
						SCM_{cv}	0.786±0.101	0.848±0.140	0.738±0.160	0.811±0.107	2.3 ± 0.9
	piperacillin/tazobac-tam	1734	1184	550	63.6	L1-logistic	0.862±0.016	0.879±0.028	0.822±0.061	0.897±0.012	19247.3 ± 14467.8
						L2-logistic	0.864±0.011	0.884±0.024	0.819±0.034	0.899±0.009	all*
						Majority	0.688±0.022	**1.000±0.000**	0.000±0.000	0.815±0.016	–
						Naive Bayes	0.766±0.015	0.759±0.015	0.782±0.025	0.817±0.014	all
						Poly-SVM	**0.886±0.014**	0.921±0.015	0.807±0.030	**0.917±0.011**	all
						RBF-SVM	**0.884±0.014**	0.921±0.014	0.804±0.027	**0.916±0.011**	all
						Random Forests	0.876±0.008	0.900±0.019	0.822±0.036	**0.909±0.008**	47680.8 ± 43218.2
						$CART_b$	0.839±0.012	0.865±0.025	0.783±0.046	0.881±0.011	9.8 ± 2.3
						$CART_{cv}$	0.842±0.014	0.873±0.029	0.775±0.080	0.884±0.010	19.7 ± 7.7
						SCM_b	0.822±0.019	0.876±0.022	0.702±0.047	0.871±0.015	4.5 ± 1.0
						SCM_{cv}	0.829±0.009	0.817±0.027	**0.854±0.067**	0.868±0.010	15.0 ± 4.5
	tetracycline	1553	799	754	56.5	L1-logistic	**0.877±0.019**	0.798±0.028	**0.966±0.018**	**0.872±0.022**	636.6 ± 66.6
						L2-logistic	0.852±0.039	0.817±0.027	0.889±0.081	0.854±0.032	all*
						Majority	0.526±0.013	**1.000±0.000**	0.000±0.000	0.689±0.011	–
						Naive Bayes	0.670±0.017	0.880±0.016	0.435±0.048	0.737±0.010	all
						Poly-SVM	0.857±0.019	0.818±0.025	0.900±0.022	0.857±0.019	all
						RBF-SVM	0.855±0.015	0.819±0.028	0.895±0.020	0.856±0.017	all
						Random Forests	**0.873±0.022**	0.796±0.027	0.958±0.029	**0.868±0.022**	31688.8 ± 26213.0
						$CART_b$	**0.872±0.017**	0.791±0.026	**0.961±0.026**	**0.866±0.018**	3.9 ± 0.3
						$CART_{cv}$	**0.878±0.013**	0.795±0.029	**0.969±0.017**	**0.872±0.016**	7.2 ± 7.8
						SCM_b	**0.871±0.018**	0.788±0.026	**0.963±0.026**	**0.865±0.020**	4.0 ± 0.0

Continued on next page

Table S1. (Continued)

Species	Antibiotic	Genomes	Resistant	Susceptible	k-mers (millions)	Method	Accuracy	Sensitivity	Specificity	F1 score	Complexity
						SCM$_{cv}$	**0.875±0.016**	0.796±0.029	**0.962±0.019**	**0.870±0.019**	8.0 ± 4.1
	ticarcillin/clavulanic acid	170	75	95	26.1	L1-logistic	0.953±0.021	**0.952±0.045**	0.959±0.039	0.948±0.029	109218.1 ± 254118.1
						L2-logistic	**0.965±0.030**	**0.952±0.060**	0.979±0.027	**0.961±0.035**	all*
						Majority	0.526±0.070	0.000±0.000	**1.000±0.000**	–	–
						Naive Bayes	0.935±0.052	**0.951±0.053**	0.924±0.083	0.932±0.054	all
						Poly-SVM	**0.962±0.028**	**0.945±0.050**	0.979±0.027	**0.958±0.032**	all
						RBF-SVM	**0.962±0.028**	**0.945±0.050**	0.979±0.027	**0.958±0.032**	all
						Random Forests	0.950±0.020	**0.946±0.041**	0.957±0.045	0.947±0.022	198.2 ± 190.5
						CART$_b$	0.909±0.040	0.896±0.086	0.930±0.063	0.903±0.041	1.0 ± 0.0
						CART$_{cv}$	0.921±0.034	0.935±0.061	0.912±0.055	0.917±0.037	2.3 ± 1.3
						SCM$_b$	0.912±0.039	0.907±0.075	0.924±0.058	0.907±0.040	1.3 ± 0.5
						SCM$_{cv}$	0.918±0.033	0.917±0.067	0.925±0.049	0.912±0.038	2.2 ± 0.6
	tobramycin	1693	964	729	64.4	L1-logistic	**0.941±0.011**	0.931±0.016	**0.955±0.021**	**0.948±0.010**	9322.4 ± 7525.6
						L2-logistic	**0.941±0.013**	0.926±0.024	**0.962±0.014**	**0.948±0.013**	all*
						Majority	0.583±0.017	**1.000±0.000**	0.000±0.000	0.737±0.014	–
						Naive Bayes	0.822±0.022	0.755±0.037	0.916±0.031	0.831±0.020	all
						Poly-SVM	0.935±0.013	0.939±0.015	0.930±0.019	0.944±0.011	all
						RBF-SVM	0.934±0.012	0.936±0.017	0.932±0.018	0.943±0.011	all
						Random Forests	**0.949±0.013**	0.944±0.016	**0.957±0.018**	**0.956±0.011**	10760.1 ± 16650.0
						CART$_b$	**0.944±0.009**	0.943±0.013	0.946±0.016	**0.952±0.008**	5.8 ± 1.8
						CART$_{cv}$	**0.947±0.011**	0.954±0.016	0.938±0.015	**0.955±0.010**	11.0 ± 4.3
						SCM$_b$	0.938±0.009	0.941±0.020	0.934±0.013	**0.947±0.009**	3.8 ± 0.6
						SCM$_{cv}$	**0.940±0.012**	0.951±0.020	0.925±0.020	**0.949±0.011**	9.1 ± 2.6
	trimethoprim	188	81	107	35.1	L1-logistic	**0.954±0.036**	0.906±0.090	0.990±0.022	**0.943±0.051**	156.7 ± 20.9
						L2-logistic	0.927±0.042	**0.914±0.061**	0.938±0.043	0.915±0.059	all*
						Majority	0.551±0.061	0.000±0.000	**1.000±0.000**	–	–
						Naive Bayes	0.611±0.120	**0.918±0.069**	0.374±0.192	0.681±0.089	all
						Poly-SVM	0.857±0.054	0.805±0.089	0.902±0.064	0.829±0.085	all
						RBF-SVM	0.854±0.051	0.793±0.094	0.907±0.062	0.824±0.082	all
						Random Forests	0.932±0.032	0.872±0.084	0.981±0.025	0.918±0.044	1702.8 ± 2378.2
						CART$_b$	0.932±0.034	0.879±0.117	0.971±0.034	0.914±0.063	1.1 ± 0.3
						CART$_{cv}$	0.932±0.029	0.873±0.096	0.974±0.036	0.915±0.049	1.6 ± 1.0
						SCM$_b$	0.938±0.026	0.902±0.074	0.967±0.032	0.926±0.038	1.0 ± 0.0
						SCM$_{cv}$	0.932±0.026	0.869±0.086	0.979±0.027	0.917±0.040	1.8 ± 0.8
	trimethoprim/sul-famethoxazole	2129	1587	542	71.3	L1-logistic	**0.935±0.011**	0.949±0.013	**0.893±0.018**	**0.956±0.008**	669.6 ± 86.5
						L2-logistic	0.924±0.013	0.942±0.018	0.871±0.041	0.949±0.010	all*
						Majority	0.752±0.026	**1.000±0.000**	0.000±0.000	0.858±0.017	–
						Naive Bayes	0.803±0.018	0.902±0.019	0.504±0.053	0.873±0.013	all
						Poly-SVM	**0.933±0.013**	0.963±0.013	0.843±0.031	**0.955±0.010**	all
						RBF-SVM	**0.932±0.013**	0.963±0.017	0.842±0.034	**0.955±0.010**	all
						Random Forests	**0.937±0.016**	0.968±0.014	0.846±0.030	**0.959±0.011**	25734.7 ± 20327.6
						CART$_b$	**0.933±0.015**	0.973±0.012	0.813±0.032	**0.956±0.011**	3.3 ± 0.5
						CART$_{cv}$	**0.932±0.015**	0.971±0.016	0.814±0.034	**0.955±0.011**	5.4 ± 2.9

Continued on next page

Table S1. (Continued)

Species	Antibiotic	Genomes	Resistant	Susceptible	k-mers (millions)	Method	Accuracy	Sensitivity	Specificity	F1 score	Complexity
						SCM_b	**0.930 ± 0.015**	0.958 ± 0.017	0.845 ± 0.056	**0.953 ± 0.011**	4.0 ± 1.6
						SCM_{cv}	**0.930 ± 0.012**	0.952 ± 0.010	0.863 ± 0.031	**0.953 ± 0.009**	9.3 ± 3.5
M. tuberculosis	amikacin	1145	208	937	7.6	L1-logistic	**0.951 ± 0.011**	**0.802 ± 0.064**	0.987 ± 0.010	0.862 ± 0.039	17781.3 ± 15688.3
						L2-logistic	0.918 ± 0.019	0.711 ± 0.064	0.970 ± 0.026	0.771 ± 0.061	all*
						Majority	0.803 ± 0.024	0.000 ± 0.000	**1.000 ± 0.000**	–	–
						Naive Bayes	0.752 ± 0.042	0.668 ± 0.098	0.773 ± 0.063	0.512 ± 0.063	all
						Poly-SVM	0.903 ± 0.021	0.613 ± 0.077	0.974 ± 0.019	0.709 ± 0.074	all
						RBF-SVM	0.902 ± 0.026	0.613 ± 0.083	0.973 ± 0.020	0.708 ± 0.085	all
						Random Forests	0.941 ± 0.010	0.748 ± 0.047	0.989 ± 0.012	0.832 ± 0.034	17558.2 ± 19697.7
						$CART_b$	**0.958 ± 0.009**	**0.808 ± 0.056**	**0.994 ± 0.009**	**0.881 ± 0.035**	1.0 ± 0.0
						$CART_{cv}$	**0.958 ± 0.009**	**0.808 ± 0.056**	**0.994 ± 0.009**	**0.881 ± 0.035**	1.0 ± 0.0
						SCM_b	**0.958 ± 0.009**	**0.808 ± 0.056**	**0.994 ± 0.009**	**0.881 ± 0.035**	1.0 ± 0.0
						SCM_{cv}	**0.958 ± 0.009**	**0.808 ± 0.056**	**0.994 ± 0.009**	**0.881 ± 0.035**	1.0 ± 0.0
	amoxicillin	766	25	741	7.3	L1-logistic	**0.981 ± 0.012**	0.585 ± 0.322	**0.993 ± 0.007**	–	1345.2 ± 331.0
						L2-logistic	0.974 ± 0.012	0.261 ± 0.243	**0.993 ± 0.005**	–	all*
						Majority	0.974 ± 0.012	0.000 ± 0.000	**1.000 ± 0.000**	–	–
						Naive Bayes	0.962 ± 0.014	0.000 ± 0.000	0.988 ± 0.007	–	all
						Poly-SVM	**0.975 ± 0.014**	0.212 ± 0.322	**0.996 ± 0.006**	–	all
						RBF-SVM	**0.975 ± 0.015**	0.187 ± 0.328	**0.996 ± 0.006**	–	all
						Random Forests	0.972 ± 0.012	0.245 ± 0.249	**0.992 ± 0.009**	–	3351.2 ± 4778.6
						$CART_b$	**0.980 ± 0.013**	0.643 ± 0.370	**0.991 ± 0.006**	–	0.9 ± 0.3
						$CART_{cv}$	**0.983 ± 0.008**	0.693 ± 0.301	**0.991 ± 0.006**	–	1.1 ± 0.3
						SCM_b	**0.984 ± 0.007**	**0.718 ± 0.294**	**0.991 ± 0.006**	–	1.0 ± 0.0
						SCM_{cv}	**0.984 ± 0.007**	0.618 ± 0.352	**0.993 ± 0.006**	–	1.4 ± 0.7
	capreomycin	1123	204	919	7.7	L1-logistic	**0.932 ± 0.020**	0.772 ± 0.056	0.971 ± 0.013	0.813 ± 0.044	34525.0 ± 17931.7
						L2-logistic	0.902 ± 0.022	0.640 ± 0.074	0.965 ± 0.019	0.712 ± 0.057	all*
						Majority	0.810 ± 0.026	0.000 ± 0.000	**1.000 ± 0.000**	–	–
						Naive Bayes	0.783 ± 0.027	0.617 ± 0.083	0.823 ± 0.023	0.517 ± 0.058	all
						Poly-SVM	0.889 ± 0.021	0.608 ± 0.063	0.955 ± 0.014	0.674 ± 0.050	all
						RBF-SVM	0.891 ± 0.022	0.601 ± 0.070	0.960 ± 0.012	0.676 ± 0.059	all
						Random Forests	0.909 ± 0.026	0.612 ± 0.077	0.980 ± 0.016	0.719 ± 0.072	15086.7 ± 18855.0
						$CART_b$	**0.938 ± 0.014**	**0.796 ± 0.065**	0.972 ± 0.011	**0.829 ± 0.035**	1.5 ± 0.5
						$CART_{cv}$	**0.938 ± 0.014**	**0.793 ± 0.075**	0.972 ± 0.014	**0.828 ± 0.040**	1.9 ± 1.0
						SCM_b	**0.938 ± 0.014**	**0.787 ± 0.062**	0.975 ± 0.010	**0.829 ± 0.034**	1.8 ± 0.4
						SCM_{cv}	**0.937 ± 0.014**	**0.791 ± 0.058**	0.972 ± 0.010	**0.826 ± 0.031**	3.5 ± 3.2
	ciprofloxacin	336	35	301	5.1	L1-logistic	0.973 ± 0.020	0.902 ± 0.132	0.979 ± 0.022	0.854 ± 0.095	734.6 ± 627.2
						L2-logistic	0.940 ± 0.031	0.650 ± 0.194	0.968 ± 0.022	0.631 ± 0.157	all*
						Majority	0.921 ± 0.022	0.000 ± 0.000	**1.000 ± 0.000**	–	–
						Naive Bayes	0.912 ± 0.022	0.039 ± 0.087	0.987 ± 0.010	–	all
						Poly-SVM	0.951 ± 0.027	0.675 ± 0.208	0.977 ± 0.020	0.684 ± 0.160	all
						RBF-SVM	0.936 ± 0.037	0.559 ± 0.289	0.972 ± 0.027	–	all
						Random Forests	0.969 ± 0.027	0.694 ± 0.248	**0.995 ± 0.008**	0.769 ± 0.211	932.2 ± 572.8
						$CART_b$	**0.982 ± 0.009**	**0.935 ± 0.106**	0.985 ± 0.009	0.888 ± 0.065	1.1 ± 0.3
						$CART_{cv}$	**0.984 ± 0.011**	0.918 ± 0.107	0.989 ± 0.011	**0.901 ± 0.072**	1.9 ± 0.9
						SCM_b	**0.982 ± 0.009**	0.918 ± 0.107	0.987 ± 0.010	0.886 ± 0.064	1.3 ± 0.5

Continued on next page

Table S1. (Continued)

Species	Antibiotic	Genomes	Resistant	Susceptible	k-mers (millions)	Method	Accuracy	Sensitivity	Specificity	F1 score	Complexity
						SCM$_{cv}$	**0.981±0.010**	0.885±0.129	0.987±0.010	0.867±0.095	1.5 ± 0.5
	cycloserine		72	264	4.8	L1-logistic	0.842±0.028	**0.618±0.109**	0.893±0.035	**0.582±0.094**	318306.1 ± 239638.1
						L2-logistic	0.839±0.034	0.461±0.188	0.919±0.043	0.491±0.172	all*
						Majority	0.815±0.045	0.000±0.000	**1.000±0.000**	–	–
						Naive Bayes	0.813±0.047	0.005±0.017	**0.996±0.009**	–	all
						Poly-SVM	0.828±0.030	0.527±0.094	0.898±0.041	0.525±0.092	all
						RBF-SVM	**0.858±0.041**	0.356±0.108	0.970±0.023	0.475±0.128	all
						Random Forests	**0.860±0.027**	0.417±0.141	0.960±0.029	0.507±0.115	8661.1 ± 11376.0
						CART$_b$	0.812±0.043	0.020±0.063	**0.993±0.022**	–	0.3 ± 0.9
						CART$_{cv}$	0.830±0.043	0.418±0.130	0.922±0.058	0.468±0.150	12.7 ± 9.5
						SCM$_b$	0.800±0.039	0.137±0.083	0.953±0.043	0.190±0.083	1.5 ± 0.5
						SCM$_{cv}$	0.822±0.040	0.302±0.152	0.942±0.056	0.365±0.104	6.5 ± 5.4
	ethambutol	4780	748	4032	11.6	L1-logistic	**0.924±0.007**	0.760±0.059	0.955±0.015	0.761±0.024	54872.7 ± 37237.7
						L2-logistic	**0.924±0.010**	**0.773±0.070**	0.952±0.013	0.762±0.033	all*
						Majority	0.841±0.006	0.000±0.000	**1.000±0.000**	–	–
						Naive Bayes	0.823±0.013	**0.769±0.053**	0.833±0.010	0.579±0.035	all
						Poly-SVM	**0.925±0.011**	0.722±0.055	0.963±0.005	0.752±0.041	all
						RBF-SVM	0.922±0.011	0.705±0.068	0.963±0.005	0.740±0.045	all
						Random Forests	**0.933±0.011**	0.752±0.045	0.967±0.006	**0.781±0.037**	55934.9 ± 51887.2
						CART$_b$	**0.926±0.010**	0.764±0.053	0.956±0.007	0.765±0.036	13.2 ± 2.1
						CART$_{cv}$	**0.924±0.012**	**0.774±0.067**	0.952±0.008	0.762±0.045	20.7 ± 8.0
						SCM$_b$	0.920±0.006	0.743±0.056	0.953±0.013	0.745±0.020	5.7 ± 1.2
						SCM$_{cv}$	0.923±0.007	**0.766±0.040**	0.952±0.011	0.758±0.018	10.2 ± 2.3
	ethionamide	564	210	354	5.0	L1-logistic	0.781±0.043	0.695±0.058	0.836±0.046	**0.709±0.052**	7671.6 ± 13150.6
						L2-logistic	0.739±0.047	**0.726±0.079**	0.746±0.104	0.681±0.043	all*
						Majority	0.616±0.032	0.000±0.000	**1.000±0.000**	–	–
						Naive Bayes	0.688±0.060	0.570±0.115	0.762±0.134	0.581±0.062	all
						Poly-SVM	0.779±0.028	0.623±0.057	0.875±0.031	0.682±0.042	all
						RBF-SVM	0.786±0.029	0.630±0.067	0.882±0.033	0.691±0.048	all
						Random Forests	**0.798±0.036**	0.592±0.073	0.925±0.041	0.691±0.054	29885.4 ± 18585.0
						CART$_b$	0.781±0.024	0.616±0.062	0.884±0.035	0.682±0.042	2.5 ± 0.5
						CART$_{cv}$	0.782±0.046	0.674±0.078	0.849±0.069	**0.703±0.062**	25.3 ± 14.0
						SCM$_b$	0.771±0.026	0.605±0.090	0.876±0.072	0.667±0.043	2.2 ± 0.4
						SCM$_{cv}$	0.762±0.032	0.601±0.092	0.863±0.091	0.658±0.042	5.0 ± 1.9
	isoniazid	5022	1719	3303	11.7	L1-logistic	**0.962±0.004**	0.921±0.012	0.984±0.005	**0.944±0.006**	2242.2 ± 202.1
						L2-logistic	0.941±0.005	0.865±0.016	0.981±0.006	0.910±0.007	all*
						Majority	0.658±0.011	0.000±0.000	**1.000±0.000**	–	–
						Naive Bayes	0.789±0.011	0.697±0.033	0.837±0.010	0.693±0.024	all
						Poly-SVM	0.934±0.007	0.845±0.019	0.980±0.005	0.897±0.011	all
						RBF-SVM	0.930±0.007	0.849±0.016	0.973±0.004	0.893±0.010	all
						Random Forests	**0.962±0.006**	0.920±0.016	0.984±0.004	**0.944±0.009**	78761.3 ± 44953.9
						CART$_b$	**0.962±0.004**	**0.935±0.011**	0.976±0.008	**0.944±0.005**	4.7 ± 1.2
						CART$_{cv}$	**0.963±0.004**	**0.943±0.010**	0.973±0.007	**0.945±0.006**	5.9 ± 2.6
						SCM$_b$	**0.963±0.005**	**0.936±0.016**	0.977±0.009	**0.945±0.007**	4.5 ± 0.5
						SCM$_{cv}$	**0.963±0.004**	**0.941±0.013**	0.975±0.008	**0.946±0.006**	5.0 ± 1.7

Continued on next page

Table S1. (Continued)

Species	Antibiotic	Genomes	Resistant	Susceptible	k-mers (millions)	Method	Accuracy	Sensitivity	Specificity	F1 score	Complexity
	kanamycin	1355	297	1058	7.6	L1-logistic	0.947 ± 0.010	**0.842 ± 0.037**	0.976 ± 0.007	0.874 ± 0.024	9464.5 ± 18157.1
						L2-logistic	0.895 ± 0.029	0.774 ± 0.060	0.929 ± 0.050	0.766 ± 0.040	all*
						Majority	0.781 ± 0.012	0.000 ± 0.000	**1.000 ± 0.000**	–	–
						Naive Bayes	0.764 ± 0.022	0.773 ± 0.027	0.762 ± 0.025	0.590 ± 0.033	all
						Poly-SVM	0.907 ± 0.010	0.714 ± 0.033	0.961 ± 0.012	0.770 ± 0.023	all
						RBF-SVM	0.911 ± 0.013	0.722 ± 0.026	0.965 ± 0.014	0.782 ± 0.026	all
						Random Forests	0.928 ± 0.010	0.754 ± 0.031	0.977 ± 0.009	0.822 ± 0.025	45035.8 ± 13637.5
						CART$_b$	**0.957 ± 0.011**	**0.844 ± 0.040**	0.989 ± 0.006	**0.895 ± 0.025**	3.0 ± 0.0
						CART$_{cv}$	**0.957 ± 0.011**	**0.844 ± 0.040**	0.989 ± 0.006	**0.895 ± 0.025**	3.0 ± 0.0
						SCM$_b$	**0.949 ± 0.012**	**0.844 ± 0.040**	0.979 ± 0.008	0.880 ± 0.028	2.0 ± 0.0
						SCM$_{cv}$	**0.949 ± 0.013**	**0.845 ± 0.038**	0.978 ± 0.010	0.879 ± 0.029	2.1 ± 0.3
	moxifloxacin	699	57	642	7.2	L1-logistic	**0.953 ± 0.017**	0.722 ± 0.123	0.976 ± 0.017	0.729 ± 0.097	6834.6 ± 7712.5
						L2-logistic	0.933 ± 0.021	0.457 ± 0.131	0.980 ± 0.013	0.543 ± 0.143	all*
						Majority	0.911 ± 0.018	0.000 ± 0.000	**1.000 ± 0.000**	–	–
						Naive Bayes	0.883 ± 0.025	0.110 ± 0.107	0.961 ± 0.024	–	all
						Poly-SVM	0.924 ± 0.012	0.231 ± 0.145	0.990 ± 0.009	–	all
						RBF-SVM	0.927 ± 0.014	0.249 ± 0.142	**0.991 ± 0.008**	–	all
						Random Forests	0.932 ± 0.014	0.328 ± 0.117	**0.991 ± 0.015**	0.453 ± 0.137	9679.0 ± 9024.3
						CART$_b$	**0.957 ± 0.020**	0.844 ± 0.162	0.968 ± 0.014	0.769 ± 0.117	1.1 ± 0.3
						CART$_{cv}$	**0.960 ± 0.014**	**0.860 ± 0.149**	0.969 ± 0.015	**0.782 ± 0.099**	1.1 ± 0.3
						SCM$_b$	0.950 ± 0.017	0.771 ± 0.185	0.969 ± 0.014	0.725 ± 0.109	1.4 ± 0.5
						SCM$_{cv}$	**0.960 ± 0.014**	**0.854 ± 0.118**	0.970 ± 0.016	**0.785 ± 0.075**	1.4 ± 1.3
	nicotinamide	167	84	83	4.6	L1-logistic	0.803 ± 0.098	0.724 ± 0.119	0.888 ± 0.123	0.791 ± 0.095	17764.3 ± 54250.8
						L2-logistic	0.730 ± 0.097	0.671 ± 0.079	0.782 ± 0.155	0.725 ± 0.076	all*
						Majority	0.433 ± 0.038	0.400 ± 0.516	0.600 ± 0.516	–	–
						Naive Bayes	0.618 ± 0.128	0.455 ± 0.271	0.826 ± 0.153	–	all
						Poly-SVM	0.752 ± 0.114	**0.783 ± 0.131**	0.715 ± 0.140	0.767 ± 0.096	all
						RBF-SVM	0.758 ± 0.111	**0.774 ± 0.137**	0.742 ± 0.113	0.769 ± 0.094	all
						Random Forests	0.736 ± 0.078	0.648 ± 0.108	0.839 ± 0.083	0.718 ± 0.076	4379.0 ± 5821.1
						CART$_b$	**0.842 ± 0.057**	0.746 ± 0.090	**0.952 ± 0.045**	**0.828 ± 0.066**	1.0 ± 0.0
						CART$_{cv}$	**0.836 ± 0.063**	0.746 ± 0.090	0.939 ± 0.058	**0.823 ± 0.069**	1.3 ± 0.7
						SCM$_b$	**0.842 ± 0.057**	0.746 ± 0.090	**0.952 ± 0.045**	**0.828 ± 0.066**	1.0 ± 0.0
						SCM$_{cv}$	0.821 ± 0.063	0.734 ± 0.099	0.919 ± 0.082	0.807 ± 0.070	2.0 ± 1.2
	ofloxacin	851	307	544	5.1	L1-logistic	**0.935 ± 0.017**	**0.888 ± 0.019**	0.963 ± 0.025	**0.912 ± 0.018**	193.9 ± 24.5
						L2-logistic	0.828 ± 0.029	0.802 ± 0.055	0.844 ± 0.037	0.776 ± 0.029	all*
						Majority	0.628 ± 0.031	0.000 ± 0.000	**1.000 ± 0.000**	–	–
						Naive Bayes	0.672 ± 0.031	0.275 ± 0.155	0.907 ± 0.067	0.357 ± 0.166	all
						Poly-SVM	0.848 ± 0.026	0.791 ± 0.046	0.883 ± 0.027	0.795 ± 0.033	all
						RBF-SVM	0.844 ± 0.025	0.782 ± 0.043	0.881 ± 0.026	0.788 ± 0.029	all
						Random Forests	0.891 ± 0.029	0.808 ± 0.052	0.940 ± 0.017	0.846 ± 0.041	33826.7 ± 23226.3
						CART$_b$	**0.938 ± 0.019**	**0.895 ± 0.020**	0.964 ± 0.026	**0.916 ± 0.021**	1.0 ± 0.0
						CART$_{cv}$	**0.938 ± 0.019**	**0.895 ± 0.020**	0.964 ± 0.026	**0.916 ± 0.021**	1.0 ± 0.0
						SCM$_b$	**0.938 ± 0.019**	**0.895 ± 0.020**	0.964 ± 0.026	**0.916 ± 0.021**	1.0 ± 0.0
						SCM$_{cv}$	**0.937 ± 0.018**	**0.895 ± 0.020**	0.962 ± 0.026	**0.914 ± 0.020**	1.4 ± 0.8
	para-aminosalicylic acid	378	80	298	4.9	L1-logistic	**0.883 ± 0.055**	0.720 ± 0.108	0.925 ± 0.051	**0.712 ± 0.127**	2944.3 ± 3479.3

Continued on next page

Table S1. (Continued)

Species	Antibiotic	Genomes	Resistant	Susceptible	k-mers (millions)	Method	Accuracy	Sensitivity	Specificity	F1 score	Complexity
						L2-logistic	0.843 ± 0.040	**0.789 ± 0.113**	0.856 ± 0.065	0.666 ± 0.070	all*
						Majority	0.797 ± 0.047	0.000 ± 0.000	**1.000 ± 0.000**	–	–
						Naive Bayes	0.856 ± 0.031	0.562 ± 0.096	0.932 ± 0.031	0.607 ± 0.085	all
						Poly-SVM	0.845 ± 0.033	0.468 ± 0.109	0.942 ± 0.044	0.543 ± 0.083	all
						RBF-SVM	0.863 ± 0.033	0.562 ± 0.133	0.940 ± 0.029	0.614 ± 0.101	all
						Random Forests	0.852 ± 0.029	0.550 ± 0.126	0.932 ± 0.036	0.592 ± 0.077	4906.8 ± 8827.5
						$CART_b$	0.835 ± 0.029	0.362 ± 0.101	0.957 ± 0.033	0.459 ± 0.090	1.0 ± 0.0
						$CART_{cv}$	0.823 ± 0.028	0.454 ± 0.116	0.918 ± 0.032	0.499 ± 0.063	10.3 ± 9.2
						SCM_b	0.836 ± 0.037	0.402 ± 0.127	0.948 ± 0.041	0.487 ± 0.110	1.1 ± 0.3
						SCM_{cv}	0.825 ± 0.035	0.418 ± 0.192	0.935 ± 0.063	0.469 ± 0.115	3.0 ± 2.7
	pyrazinamide	3668	377	3291	10.6	L1-logistic	**0.944 ± 0.009**	**0.696 ± 0.064**	0.971 ± 0.007	**0.707 ± 0.043**	63589.6 ± 18666.8
						L2-logistic	**0.938 ± 0.008**	**0.695 ± 0.069**	0.965 ± 0.005	0.685 ± 0.036	all*
						Majority	0.903 ± 0.009	0.000 ± 0.000	**1.000 ± 0.000**	–	–
						Naive Bayes	0.842 ± 0.016	0.673 ± 0.070	0.860 ± 0.017	0.451 ± 0.039	all
						Poly-SVM	**0.942 ± 0.008**	0.665 ± 0.062	0.972 ± 0.005	0.689 ± 0.036	all
						RBF-SVM	**0.941 ± 0.008**	0.658 ± 0.057	0.971 ± 0.005	0.682 ± 0.033	all
						Random Forests	**0.944 ± 0.009**	0.633 ± 0.072	0.977 ± 0.007	0.685 ± 0.047	43384.9 ± 32114.6
						$CART_b$	**0.942 ± 0.012**	0.609 ± 0.067	0.978 ± 0.008	0.671 ± 0.054	11.3 ± 2.0
						$CART_{cv}$	**0.945 ± 0.009**	0.584 ± 0.060	0.984 ± 0.009	0.671 ± 0.038	17.4 ± 9.4
						SCM_b	**0.943 ± 0.008**	0.571 ± 0.056	0.983 ± 0.006	0.657 ± 0.038	7.6 ± 1.6
						SCM_{cv}	**0.941 ± 0.010**	0.613 ± 0.046	0.977 ± 0.009	0.669 ± 0.038	13.4 ± 4.0
	rifabutin	161	72	89	4.7	L1-logistic	**0.828 ± 0.045**	0.795 ± 0.073	0.848 ± 0.094	0.814 ± 0.041	47.9 ± 11.6
						L2-logistic	0.619 ± 0.062	0.621 ± 0.191	0.616 ± 0.123	0.593 ± 0.125	all*
						Majority	0.522 ± 0.078	0.000 ± 0.000	**1.000 ± 0.000**	–	–
						Naive Bayes	0.575 ± 0.082	0.584 ± 0.148	0.574 ± 0.096	0.559 ± 0.111	all
						Poly-SVM	0.641 ± 0.068	0.589 ± 0.114	0.690 ± 0.064	0.605 ± 0.082	all
						RBF-SVM	0.631 ± 0.073	0.572 ± 0.103	0.684 ± 0.089	0.593 ± 0.087	all
						Random Forests	0.678 ± 0.096	0.555 ± 0.139	0.793 ± 0.132	0.616 ± 0.114	7461.1 ± 7864.9
						$CART_b$	**0.834 ± 0.047**	**0.819 ± 0.071**	0.835 ± 0.085	**0.824 ± 0.047**	1.0 ± 0.0
						$CART_{cv}$	**0.828 ± 0.054**	**0.813 ± 0.067**	0.829 ± 0.088	**0.818 ± 0.052**	1.7 ± 1.6
						SCM_b	**0.834 ± 0.047**	**0.819 ± 0.071**	0.835 ± 0.085	**0.824 ± 0.047**	1.0 ± 0.0
						SCM_{cv}	**0.825 ± 0.040**	**0.811 ± 0.054**	0.822 ± 0.095	**0.815 ± 0.035**	1.5 ± 0.7
	rifampin	5022	1396	3626	11.7	L1-logistic	**0.974 ± 0.005**	**0.962 ± 0.013**	0.979 ± 0.005	**0.954 ± 0.009**	1376.3 ± 164.7
						L2-logistic	0.958 ± 0.008	0.902 ± 0.014	0.979 ± 0.007	0.922 ± 0.014	all*
						Majority	0.724 ± 0.011	0.000 ± 0.000	**1.000 ± 0.000**	–	–
						Naive Bayes	0.828 ± 0.011	0.821 ± 0.026	0.831 ± 0.011	0.725 ± 0.021	all
						Poly-SVM	0.950 ± 0.007	0.883 ± 0.014	0.976 ± 0.006	0.907 ± 0.013	all
						RBF-SVM	0.948 ± 0.009	0.885 ± 0.015	0.972 ± 0.007	0.904 ± 0.015	all
						Random Forests	0.965 ± 0.005	0.932 ± 0.011	0.978 ± 0.006	0.937 ± 0.008	77974.3 ± 44091.8
						$CART_b$	**0.977 ± 0.005**	**0.963 ± 0.014**	0.982 ± 0.005	**0.958 ± 0.009**	4.0 ± 0.9
						$CART_{cv}$	**0.978 ± 0.005**	**0.966 ± 0.014**	0.982 ± 0.006	**0.960 ± 0.008**	4.6 ± 1.1
						SCM_b	**0.977 ± 0.005**	**0.963 ± 0.014**	0.982 ± 0.005	**0.958 ± 0.009**	3.4 ± 0.5
						SCM_{cv}	**0.977 ± 0.005**	**0.966 ± 0.013**	0.982 ± 0.006	**0.960 ± 0.008**	4.2 ± 1.0
	streptomycin	3406	1084	2322	9.9	L1-logistic	**0.907 ± 0.004**	**0.865 ± 0.015**	0.926 ± 0.007	**0.854 ± 0.007**	1926.9 ± 130.3
						L2-logistic	0.895 ± 0.008	0.817 ± 0.017	0.931 ± 0.009	0.830 ± 0.015	all*

Continued on next page

Table S1. (Continued)

Species	Antibiotic	Genomes	Resistant	Susceptible	*k*-mers (millions)	Method	Accuracy	Sensitivity	Specificity	F1 score	Complexity
						Majority	0.687±0.009	0.000±0.000	**1.000±0.000**	–	–
						Naive Bayes	0.761±0.020	0.713±0.030	0.783±0.025	0.652±0.026	all
						Poly-SVM	0.896±0.008	0.797±0.025	0.941±0.010	0.827±0.016	all
						RBF-SVM	0.892±0.009	0.780±0.025	0.943±0.012	0.818±0.018	all
						Random Forests	**0.906±0.008**	0.805±0.024	0.952±0.007	0.843±0.016	68247.2 ± 46223.9
						CART$_b$	**0.910±0.006**	0.805±0.027	0.958±0.011	**0.848±0.011**	10.0 ± 1.4
						CART$_{cv}$	**0.907±0.006**	0.807±0.032	0.953±0.014	**0.845±0.012**	17.5 ± 11.8
						SCM$_b$	**0.906±0.010**	0.783±0.037	0.961±0.012	0.838±0.019	6.8 ± 0.9
						SCM$_{cv}$	**0.908±0.011**	0.777±0.029	0.968±0.009	0.841±0.021	11.2 ± 3.5
N. gonorrhoeae	azithromycin	392	214	178	4.8	L1-logistic	**0.942±0.024**	0.939±0.036	**0.945±0.039**	**0.945±0.025**	6095.6 ± 9342.0
						L2-logistic	0.915±0.031	0.903±0.048	0.928±0.032	0.918±0.030	all*
						Majority	0.529±0.035	**1.000±0.000**	0.000±0.000	0.692±0.030	–
						Naive Bayes	0.736±0.055	0.596±0.086	0.894±0.045	0.702±0.072	all
						Poly-SVM	0.906±0.038	0.902±0.057	0.910±0.046	0.909±0.038	all
						RBF-SVM	0.905±0.035	0.902±0.053	0.907±0.040	0.908±0.035	all
						Random Forests	0.895±0.040	0.893±0.049	0.897±0.045	0.899±0.039	4571.7 ± 7185.8
						CART$_b$	**0.936±0.039**	0.969±0.028	0.899±0.057	**0.942±0.035**	3.3 ± 0.5
						CART$_{cv}$	0.929±0.031	0.962±0.030	0.894±0.047	**0.935±0.028**	6.1 ± 3.8
						SCM$_b$	**0.935±0.030**	0.974±0.023	0.891±0.047	**0.941±0.026**	3.0 ± 0.0
						SCM$_{cv}$	**0.935±0.033**	0.972±0.024	0.894±0.048	**0.940±0.029**	3.5 ± 0.8
	ciprofloxacin	173	106	67	3.0	L1-logistic	**0.971±0.024**	0.974±0.037	0.967±0.060	**0.977±0.018**	9440.5 ± 24435.6
						L2-logistic	**0.968±0.017**	0.950±0.024	**1.000±0.000**	**0.974±0.013**	all*
						Majority	0.638±0.048	**1.000±0.000**	0.000±0.000	0.778±0.036	–
						Naive Bayes	0.935±0.053	0.899±0.086	**1.000±0.000**	0.945±0.049	all
						Poly-SVM	**0.971±0.020**	0.955±0.029	**1.000±0.000**	**0.977±0.015**	all
						RBF-SVM	**0.971±0.014**	0.954±0.021	**1.000±0.000**	**0.977±0.011**	all
						Random Forests	**0.968±0.035**	0.965±0.050	0.976±0.039	**0.975±0.027**	1026.2 ± 1402.6
						CART$_b$	**0.971±0.031**	**0.991±0.029**	0.935±0.065	**0.977±0.024**	1.0 ± 0.0
						CART$_{cv}$	0.956±0.040	0.977±0.038	0.917±0.070	0.966±0.030	1.1 ± 0.3
						SCM$_b$	**0.971±0.031**	**0.991±0.029**	0.935±0.065	**0.977±0.024**	1.0 ± 0.0
						SCM$_{cv}$	**0.965±0.030**	0.982±0.032	0.935±0.065	**0.973±0.024**	1.1 ± 0.3
	erythromycin	178	97	81		L1-logistic	0.869±0.041	0.887±0.064	**0.838±0.082**	0.882±0.041	130.7 ± 13.7
						L2-logistic	0.849±0.036	0.866±0.071	0.818±0.074	0.864±0.039	all*
						Majority	0.566±0.061	**1.000±0.000**	0.000±0.000	0.721±0.051	–
						Naive Bayes	0.843±0.078	0.831±0.116	**0.846±0.076**	0.850±0.086	all
						Poly-SVM	0.869±0.047	0.902±0.064	0.818±0.097	0.885±0.041	all
						RBF-SVM	0.866±0.049	0.902±0.064	0.811±0.090	0.882±0.044	all
						Random Forests	0.871±0.036	0.909±0.038	0.818±0.066	0.889±0.029	413.5 ± 822.4
						CART$_b$	**0.883±0.041**	0.919±0.047	0.831±0.075	**0.898±0.034**	1.0 ± 0.0
						CART$_{cv}$	**0.886±0.038**	0.925±0.047	0.831±0.075	**0.901±0.030**	1.0 ± 0.0
						SCM$_b$	**0.889±0.044**	0.925±0.047	**0.838±0.082**	**0.904±0.035**	1.0 ± 0.0
						SCM$_{cv}$	0.874±0.049	0.908±0.055	0.825±0.079	0.889±0.045	1.2 ± 0.6
	tetracycline	142	109	33	2.9	L1-logistic	0.929±0.038	0.972±0.048	0.758±0.180	0.954±0.026	40683.8 ± 76894.8
						L2-logistic	0.904±0.058	0.929±0.065	**0.801±0.170**	0.938±0.036	all*
						Majority	0.775±0.073	**1.000±0.000**	0.000±0.000	0.872±0.047	–

Continued on next page

Table S1. (Continued)

Species	Antibiotic	Genomes	Resistant	Susceptible	*k*-mers (millions)	Method	Accuracy	Sensitivity	Specificity	F1 score	Complexity
						Naive Bayes	0.896 ± 0.064	0.920 ± 0.058	**0.801 ± 0.170**	0.933 ± 0.039	all
						Poly-SVM	**0.950 ± 0.038**	**0.996 ± 0.013**	0.770 ± 0.177	**0.969 ± 0.023**	all
						RBF-SVM	**0.950 ± 0.038**	**0.996 ± 0.013**	0.770 ± 0.177	**0.969 ± 0.023**	all
						Random Forests	0.936 ± 0.060	0.969 ± 0.053	**0.801 ± 0.170**	0.959 ± 0.036	1010.6 ± 1560.9
						CART$_b$	0.918 ± 0.053	0.966 ± 0.044	0.747 ± 0.190	0.949 ± 0.033	1.0 ± 0.0
						CART$_{cv}$	0.896 ± 0.043	0.940 ± 0.074	0.736 ± 0.184	0.932 ± 0.034	1.5 ± 0.8
						SCM$_b$	0.896 ± 0.059	0.942 ± 0.072	0.735 ± 0.175	0.933 ± 0.040	1.0 ± 0.0
						SCM$_{cv}$	0.907 ± 0.042	0.950 ± 0.062	0.747 ± 0.190	0.940 ± 0.029	1.3 ± 0.5
P. aeruginosa	amikacin	498	90	408	43.2	L1-logistic	0.879 ± 0.029	0.576 ± 0.095	0.942 ± 0.024	0.620 ± 0.097	33987.3 ± 66238.1
						L2-logistic	0.845 ± 0.030	0.553 ± 0.127	0.908 ± 0.026	0.550 ± 0.092	all*
						Majority	0.824 ± 0.031	0.000 ± 0.000	**1.000 ± 0.000**	–	–
						Naive Bayes	0.802 ± 0.030	**0.630 ± 0.090**	0.838 ± 0.027	0.523 ± 0.088	all
						Poly-SVM	0.848 ± 0.031	0.417 ± 0.136	0.941 ± 0.024	0.479 ± 0.122	all
						RBF-SVM	0.864 ± 0.028	0.414 ± 0.101	0.960 ± 0.024	0.509 ± 0.108	all
						Random Forests	0.874 ± 0.023	0.536 ± 0.092	0.947 ± 0.009	0.594 ± 0.069	12334.6 ± 9968.8
						CART$_b$	0.860 ± 0.041	0.422 ± 0.172	0.953 ± 0.027	0.499 ± 0.150	2.7 ± 0.9
						CART$_{cv}$	0.861 ± 0.037	0.482 ± 0.150	0.944 ± 0.034	0.539 ± 0.116	6.8 ± 2.8
						SCM$_b$	**0.891 ± 0.022**	0.604 ± 0.134	0.953 ± 0.021	0.650 ± 0.098	3.6 ± 0.5
						SCM$_{cv}$	**0.888 ± 0.026**	**0.638 ± 0.098**	0.940 ± 0.023	**0.661 ± 0.101**	4.6 ± 1.3
	ciprofloxacin	132	29	103	22.5	L1-logistic	**0.969 ± 0.030**	0.883 ± 0.150	**0.994 ± 0.018**	**0.926 ± 0.089**	381.5 ± 452.3
						L2-logistic	0.808 ± 0.091	0.412 ± 0.134	0.944 ± 0.048	0.519 ± 0.141	all*
						Majority	0.742 ± 0.115	0.000 ± 0.000	**1.000 ± 0.000**	–	–
						Naive Bayes	0.708 ± 0.100	0.385 ± 0.178	0.810 ± 0.092	–	all
						Poly-SVM	0.788 ± 0.125	0.287 ± 0.237	0.985 ± 0.033	–	all
						RBF-SVM	0.792 ± 0.125	0.304 ± 0.246	0.985 ± 0.033	–	all
						Random Forests	0.823 ± 0.095	0.396 ± 0.166	0.978 ± 0.029	0.527 ± 0.151	1515.5 ± 2132.5
						CART$_b$	**0.965 ± 0.038**	**0.917 ± 0.133**	0.982 ± 0.029	**0.934 ± 0.086**	1.0 ± 0.0
						CART$_{cv}$	**0.962 ± 0.036**	0.883 ± 0.150	0.982 ± 0.029	0.914 ± 0.092	1.0 ± 0.0
						SCM$_b$	0.958 ± 0.034	0.867 ± 0.145	0.982 ± 0.029	0.905 ± 0.087	1.0 ± 0.0
						SCM$_{cv}$	0.958 ± 0.034	0.867 ± 0.145	0.982 ± 0.029	0.905 ± 0.087	1.0 ± 0.0
	levofloxacin	491	201	290	43.0	L1-logistic	**0.937 ± 0.024**	0.893 ± 0.046	0.967 ± 0.033	0.921 ± 0.029	87.8 ± 9.6
						L2-logistic	0.828 ± 0.043	0.789 ± 0.077	0.855 ± 0.048	0.789 ± 0.060	all*
						Majority	0.588 ± 0.027	0.000 ± 0.000	**1.000 ± 0.000**	–	–
						Naive Bayes	0.768 ± 0.051	0.666 ± 0.108	0.842 ± 0.046	0.700 ± 0.078	all
						Poly-SVM	0.773 ± 0.050	0.669 ± 0.073	0.848 ± 0.058	0.708 ± 0.066	all
						RBF-SVM	0.762 ± 0.041	0.643 ± 0.103	0.846 ± 0.055	0.687 ± 0.072	all
						Random Forests	0.874 ± 0.035	0.812 ± 0.083	0.918 ± 0.040	0.840 ± 0.051	21600.5 ± 14329.4
						CART$_b$	**0.942 ± 0.028**	0.926 ± 0.037	0.952 ± 0.038	**0.931 ± 0.031**	1.1 ± 0.3
						CART$_{cv}$	**0.941 ± 0.021**	**0.963 ± 0.026**	0.924 ± 0.037	**0.932 ± 0.020**	2.5 ± 1.1
						SCM$_b$	**0.939 ± 0.023**	0.929 ± 0.041	0.945 ± 0.034	**0.927 ± 0.025**	1.2 ± 0.4
						SCM$_{cv}$	**0.939 ± 0.028**	0.917 ± 0.048	0.954 ± 0.039	**0.926 ± 0.033**	1.4 ± 0.5
	meropenem	380	163	217	39.0	L1-logistic	**0.720 ± 0.047**	0.625 ± 0.107	0.785 ± 0.043	0.646 ± 0.085	3827.0 ± 7601.6
						L2-logistic	0.688 ± 0.035	0.586 ± 0.079	0.761 ± 0.046	0.608 ± 0.060	all*
						Majority	0.583 ± 0.035	0.000 ± 0.000	**1.000 ± 0.000**	–	–
						Naive Bayes	0.663 ± 0.036	0.546 ± 0.057	0.746 ± 0.057	0.573 ± 0.059	all

Continued on next page

Table S1. (Continued)

Species	Antibiotic	Genomes	Resistant	Susceptible	k-mers (millions)	Method	Accuracy	Sensitivity	Specificity	F1 score	Complexity
						Poly-SVM	0.688±0.047	0.536±0.105	0.798±0.075	0.585±0.080	all
						RBF-SVM	0.679±0.038	0.535±0.081	0.781±0.080	0.579±0.058	all
						Random Forests	**0.724±0.035**	0.608±0.059	0.805±0.059	0.646±0.046	8561.0 ± 9899.8
						$CART_b$	**0.724±0.040**	**0.650±0.099**	0.778±0.069	**0.659±0.055**	1.1 ± 0.3
						$CART_{cv}$	0.711±0.038	**0.647±0.106**	0.757±0.072	0.647±0.067	2.6 ± 3.9
						SCM_b	**0.722±0.038**	**0.650±0.099**	0.776±0.067	**0.658±0.055**	1.2 ± 0.4
						SCM_{cv}	0.700±0.038	0.619±0.131	0.762±0.079	0.626±0.073	4.1 ± 6.0
P. difficile	azithromycin	461	213	248	19.8	L1-logistic	0.947±0.020	0.934±0.037	0.958±0.026	0.941±0.023	52144.5 ± 97222.7
						L2-logistic	0.940±0.024	0.936±0.034	0.944±0.031	0.934±0.028	all*
						Majority	0.543±0.027	0.000±0.000	**1.000±0.000**	–	–
						Naive Bayes	0.864±0.036	0.768±0.051	0.946±0.031	0.838±0.041	all
						Poly-SVM	0.951±0.023	0.943±0.038	0.959±0.026	0.946±0.028	all
						RBF-SVM	0.947±0.025	0.938±0.038	0.955±0.031	0.941±0.030	all
						Random Forests	0.942±0.016	0.929±0.035	0.955±0.027	0.936±0.020	794.1 ± 802.7
						$CART_b$	**0.985±0.009**	**0.981±0.010**	0.988±0.014	**0.983±0.011**	3.0 ± 0.0
						$CART_{cv}$	**0.976±0.017**	0.965±0.029	0.986±0.013	**0.974±0.019**	3.9 ± 1.4
						SCM_b	**0.978±0.014**	0.967±0.032	0.988±0.014	**0.976±0.016**	3.0 ± 0.7
						SCM_{cv}	**0.984±0.011**	**0.979±0.013**	0.988±0.014	**0.982±0.012**	3.3 ± 0.5
	ceftriaxone	212	150	62	11.1	L1-logistic	0.902±0.038	0.936±0.046	0.809±0.161	**0.934±0.026**	101937.2 ± 234203.7
						L2-logistic	0.907±0.029	0.933±0.041	0.844±0.145	**0.936±0.020**	all*
						Majority	0.743±0.055	**1.000±0.000**	0.000±0.000	0.851±0.036	–
						Naive Bayes	0.824±0.036	0.792±0.046	**0.921±0.062**	0.869±0.031	all
						Poly-SVM	0.895±0.026	0.930±0.045	0.798±0.137	0.929±0.019	all
						RBF-SVM	0.905±0.034	0.942±0.045	0.798±0.137	**0.936±0.024**	all
						Random Forests	**0.917±0.034**	0.939±0.042	0.858±0.155	**0.943±0.023**	436.1 ± 474.3
						$CART_b$	0.886±0.029	0.914±0.057	0.822±0.177	0.921±0.023	1.3 ± 0.5
						$CART_{cv}$	0.890±0.044	0.923±0.060	0.810±0.177	0.925±0.032	2.0 ± 1.2
						SCM_b	0.893±0.036	0.929±0.063	0.793±0.164	0.927±0.028	1.2 ± 0.4
						SCM_{cv}	0.890±0.038	0.921±0.056	0.820±0.175	0.925±0.028	1.7 ± 0.8
	clarithromycin	461	213	248	19.8	L1-logistic	0.941±0.019	0.936±0.044	0.946±0.032	0.935±0.022	153841.5 ± 267155.4
						L2-logistic	0.936±0.018	0.924±0.050	0.946±0.036	0.929±0.020	all*
						Majority	0.543±0.027	0.000±0.000	**1.000±0.000**	–	–
						Naive Bayes	0.857±0.028	0.748±0.046	0.948±0.018	0.826±0.033	all
						Poly-SVM	0.947±0.021	0.932±0.053	0.960±0.028	0.941±0.023	all
						RBF-SVM	0.945±0.024	0.927±0.057	0.960±0.028	0.938±0.027	all
						Random Forests	0.937±0.018	0.924±0.045	0.948±0.026	0.930±0.021	4726.5 ± 4502.3
						$CART_b$	**0.972±0.022**	0.970±0.048	0.974±0.010	**0.969±0.025**	2.9 ± 0.3
						$CART_{cv}$	**0.977±0.008**	**0.981±0.019**	0.974±0.010	**0.975±0.009**	3.0 ± 0.0
						SCM_b	**0.972±0.022**	0.970±0.048	0.974±0.010	**0.969±0.025**	2.9 ± 0.3
						SCM_{cv}	**0.977±0.008**	**0.981±0.019**	0.974±0.010	**0.975±0.009**	3.0 ± 0.0
	clindamycin	265	34	231	17.8	L1-logistic	**0.998±0.006**	0.989±0.035	**1.000±0.000**	**0.994±0.019**	1153.1 ± 828.3
						L2-logistic	0.974±0.020	0.889±0.107	0.986±0.017	0.904±0.060	all*
						Majority	0.872±0.057	0.000±0.000	**1.000±0.000**	–	–
						Naive Bayes	0.734±0.043	**1.000±0.000**	0.695±0.047	0.473±0.120	all
						Poly-SVM	0.964±0.017	0.877±0.124	0.978±0.016	0.859±0.041	all

Continued on next page

Table S1. (Continued)

Species	Antibiotic	Genomes	Resistant	Susceptible	k-mers (millions)	Method	Accuracy	Sensitivity	Specificity	F1 score	Complexity
						RBF-SVM	0.960 ± 0.023	0.889 ± 0.107	0.971 ± 0.021	0.854 ± 0.043	all
						Random Forests	**0.994 ± 0.009**	0.963 ± 0.059	**1.000 ± 0.000**	0.981 ± 0.032	60.1 ± 77.0
						$CART_b$	0.972 ± 0.020	0.931 ± 0.112	0.981 ± 0.016	0.888 ± 0.077	1.8 ± 0.4
						$CART_{cv}$	0.975 ± 0.022	0.931 ± 0.112	0.985 ± 0.015	0.913 ± 0.067	1.6 ± 0.5
						SCM_b	0.975 ± 0.022	0.967 ± 0.075	0.978 ± 0.014	0.904 ± 0.083	2.0 ± 0.0
						SCM_{cv}	0.975 ± 0.022	0.931 ± 0.112	0.985 ± 0.015	0.913 ± 0.067	1.6 ± 0.5
	moxifloxacin	462	188	274	19.8	L1-logistic	0.957 ± 0.027	0.921 ± 0.040	0.980 ± 0.038	0.944 ± 0.033	121.8 ± 12.6
						L2-logistic	0.936 ± 0.020	0.907 ± 0.042	0.955 ± 0.029	0.918 ± 0.028	all*
						Majority	0.599 ± 0.029	0.000 ± 0.000	**1.000 ± 0.000**	–	–
						Naive Bayes	0.887 ± 0.035	0.820 ± 0.065	0.931 ± 0.042	0.852 ± 0.048	all
						Poly-SVM	0.949 ± 0.014	0.904 ± 0.044	0.978 ± 0.022	0.934 ± 0.020	all
						RBF-SVM	0.951 ± 0.014	0.898 ± 0.048	0.985 ± 0.018	0.935 ± 0.023	all
						Random Forests	0.949 ± 0.015	0.904 ± 0.044	0.978 ± 0.022	0.934 ± 0.021	662.2 ± 669.2
						$CART_b$	**0.982 ± 0.009**	**0.959 ± 0.023**	**0.996 ± 0.008**	**0.976 ± 0.012**	1.0 ± 0.0
						$CART_{cv}$	**0.982 ± 0.009**	**0.959 ± 0.023**	**0.996 ± 0.008**	**0.976 ± 0.012**	1.1 ± 0.3
						SCM_b	**0.982 ± 0.009**	**0.959 ± 0.023**	**0.996 ± 0.008**	**0.976 ± 0.012**	1.0 ± 0.0
						SCM_{cv}	**0.982 ± 0.009**	**0.959 ± 0.023**	**0.996 ± 0.008**	**0.976 ± 0.012**	1.0 ± 0.0
S. aureus	ciprofloxacin	1229	467	762	12.3	L1-logistic	**0.983 ± 0.008**	**0.967 ± 0.015**	**0.994 ± 0.005**	**0.978 ± 0.011**	912.2 ± 1731.0
						L2-logistic	**0.975 ± 0.011**	**0.962 ± 0.022**	0.984 ± 0.011	0.969 ± 0.014	all*
						Majority	0.598 ± 0.021	0.000 ± 0.000	**1.000 ± 0.000**	–	–
						Naive Bayes	0.892 ± 0.009	0.812 ± 0.020	0.945 ± 0.013	0.858 ± 0.011	all
						Poly-SVM	**0.976 ± 0.011**	**0.960 ± 0.019**	0.986 ± 0.009	0.969 ± 0.013	all
						RBF-SVM	**0.976 ± 0.010**	**0.960 ± 0.017**	0.988 ± 0.009	**0.970 ± 0.012**	all
						Random Forests	**0.976 ± 0.010**	0.956 ± 0.023	0.989 ± 0.003	0.969 ± 0.012	16134.3 ± 13601.7
						$CART_b$	**0.983 ± 0.007**	**0.965 ± 0.014**	**0.996 ± 0.004**	**0.979 ± 0.008**	1.0 ± 0.0
						$CART_{cv}$	**0.983 ± 0.006**	**0.967 ± 0.015**	**0.994 ± 0.006**	**0.978 ± 0.008**	1.3 ± 0.7
						SCM_b	**0.983 ± 0.007**	**0.965 ± 0.014**	**0.996 ± 0.004**	**0.979 ± 0.008**	1.0 ± 0.0
						SCM_{cv}	**0.983 ± 0.006**	**0.965 ± 0.014**	**0.995 ± 0.003**	**0.978 ± 0.008**	1.2 ± 0.4
	clindamycin	624	350	274	9.6	L1-logistic	**0.969 ± 0.013**	0.978 ± 0.017	**0.955 ± 0.034**	**0.972 ± 0.012**	710.4 ± 968.6
						L2-logistic	0.957 ± 0.013	0.962 ± 0.029	**0.949 ± 0.025**	0.962 ± 0.014	all*
						Majority	0.566 ± 0.045	**1.000 ± 0.000**	0.000 ± 0.000	0.722 ± 0.039	–
						Naive Bayes	0.866 ± 0.036	0.888 ± 0.039	0.836 ± 0.052	0.882 ± 0.029	all
						Poly-SVM	0.949 ± 0.017	0.951 ± 0.038	0.944 ± 0.028	0.954 ± 0.021	all
						RBF-SVM	0.950 ± 0.011	0.954 ± 0.030	0.942 ± 0.026	0.955 ± 0.015	all
						Random Forests	**0.961 ± 0.014**	0.966 ± 0.026	**0.953 ± 0.033**	**0.966 ± 0.012**	3976.7 ± 4930.6
						$CART_b$	**0.961 ± 0.014**	0.972 ± 0.025	**0.946 ± 0.033**	**0.965 ± 0.013**	2.6 ± 1.3
						$CART_{cv}$	0.958 ± 0.008	0.965 ± 0.022	**0.947 ± 0.030**	**0.963 ± 0.008**	4.4 ± 2.2
						SCM_b	**0.961 ± 0.016**	0.971 ± 0.020	**0.947 ± 0.035**	**0.966 ± 0.014**	2.0 ± 0.0
						SCM_{cv}	**0.961 ± 0.016**	0.971 ± 0.020	**0.947 ± 0.035**	**0.966 ± 0.014**	2.2 ± 0.4
	erythromycin	1305	484	821	12.4	L1-logistic	**0.976 ± 0.009**	**0.978 ± 0.012**	0.976 ± 0.016	**0.970 ± 0.012**	10563.3 ± 27868.0
						L2-logistic	**0.976 ± 0.006**	**0.977 ± 0.008**	0.976 ± 0.009	**0.970 ± 0.007**	all*
						Majority	0.611 ± 0.019	0.000 ± 0.000	**1.000 ± 0.000**	–	–
						Naive Bayes	0.764 ± 0.027	0.772 ± 0.060	0.759 ± 0.024	0.717 ± 0.041	all
						Poly-SVM	**0.975 ± 0.010**	**0.979 ± 0.010**	0.973 ± 0.016	**0.968 ± 0.013**	all
						RBF-SVM	**0.973 ± 0.010**	**0.975 ± 0.009**	0.972 ± 0.018	**0.966 ± 0.013**	all

Continued on next page

Table S1. (Continued)

Species	Antibiotic	Genomes	Resistant	Susceptible	k-mers (millions)	Method	Accuracy	Sensitivity	Specificity	F1 score	Complexity
						Random Forests	**0.969 ± 0.010**	**0.978 ± 0.007**	0.963 ± 0.017	**0.961 ± 0.012**	6113.3 ± 7868.4
						$CART_b$	**0.976 ± 0.009**	**0.975 ± 0.009**	0.976 ± 0.016	**0.969 ± 0.012**	3.0 ± 0.0
						$CART_{cv}$	**0.974 ± 0.008**	**0.975 ± 0.011**	0.974 ± 0.016	**0.967 ± 0.011**	3.6 ± 1.3
						SCM_b	**0.976 ± 0.010**	**0.977 ± 0.008**	0.976 ± 0.016	**0.970 ± 0.012**	3.0 ± 0.0
						SCM_{cv}	**0.973 ± 0.012**	**0.975 ± 0.006**	0.972 ± 0.020	**0.966 ± 0.015**	4.6 ± 2.1
	fusidic acid	986	82	904	11.9	L1-logistic	**0.984 ± 0.009**	0.844 ± 0.117	**0.997 ± 0.003**	0.896 ± 0.068	3120.5 ± 947.0
						L2-logistic	0.969 ± 0.012	0.713 ± 0.152	**0.994 ± 0.005**	0.793 ± 0.092	all*
						Majority	0.911 ± 0.019	0.000 ± 0.000	**1.000 ± 0.000**	–	–
						Naive Bayes	0.675 ± 0.082	0.767 ± 0.122	0.664 ± 0.092	0.301 ± 0.065	all
						Poly-SVM	0.968 ± 0.015	0.686 ± 0.153	**0.995 ± 0.006**	0.780 ± 0.112	all
						RBF-SVM	0.969 ± 0.015	0.712 ± 0.167	**0.994 ± 0.005**	0.793 ± 0.108	all
						Random Forests	**0.975 ± 0.014**	0.732 ± 0.145	**0.999 ± 0.002**	0.832 ± 0.095	4322.9 ± 7941.6
						$CART_b$	**0.976 ± 0.011**	0.811 ± 0.135	**0.991 ± 0.005**	0.843 ± 0.089	2.5 ± 0.5
						$CART_{cv}$	**0.984 ± 0.010**	**0.917 ± 0.077**	**0.991 ± 0.005**	**0.907 ± 0.053**	3.7 ± 0.9
						SCM_b	**0.979 ± 0.011**	0.855 ± 0.114	**0.991 ± 0.005**	0.871 ± 0.068	2.7 ± 0.5
						SCM_{cv}	**0.983 ± 0.010**	**0.917 ± 0.077**	0.990 ± 0.006	**0.904 ± 0.054**	3.2 ± 0.6
	gentamicin	1306	162	1144	12.4	L1-logistic	**0.997 ± 0.003**	**0.981 ± 0.018**	**0.999 ± 0.002**	**0.985 ± 0.013**	136.0 ± 309.3
						L2-logistic	**0.993 ± 0.005**	0.945 ± 0.053	**0.999 ± 0.003**	0.966 ± 0.031	all*
						Majority	0.874 ± 0.019	0.000 ± 0.000	**1.000 ± 0.000**	–	–
						Naive Bayes	0.949 ± 0.038	0.906 ± 0.061	0.954 ± 0.042	0.826 ± 0.104	all
						Poly-SVM	**0.989 ± 0.006**	0.921 ± 0.056	**0.998 ± 0.003**	0.952 ± 0.032	all
						RBF-SVM	**0.990 ± 0.006**	0.921 ± 0.056	**0.999 ± 0.002**	0.953 ± 0.033	all
						Random Forests	**0.995 ± 0.004**	0.968 ± 0.043	**0.999 ± 0.002**	**0.979 ± 0.024**	432.2 ± 714.2
						$CART_b$	**0.996 ± 0.003**	**0.975 ± 0.019**	**0.999 ± 0.002**	**0.983 ± 0.012**	1.0 ± 0.0
						$CART_{cv}$	**0.996 ± 0.003**	**0.975 ± 0.019**	**0.999 ± 0.002**	**0.983 ± 0.012**	1.0 ± 0.0
						SCM_b	**0.996 ± 0.003**	**0.975 ± 0.019**	**0.999 ± 0.002**	**0.983 ± 0.012**	1.0 ± 0.0
						SCM_{cv}	**0.994 ± 0.004**	0.967 ± 0.027	**0.998 ± 0.002**	**0.977 ± 0.016**	1.2 ± 0.4
	methicillin	1593	707	886	13.3	L1-logistic	**0.988 ± 0.005**	**0.985 ± 0.010**	**0.991 ± 0.007**	**0.987 ± 0.005**	230.6 ± 212.3
						L2-logistic	**0.987 ± 0.003**	**0.984 ± 0.010**	0.990 ± 0.007	**0.986 ± 0.003**	all*
						Majority	0.544 ± 0.016	0.000 ± 0.000	**1.000 ± 0.000**	–	–
						Naive Bayes	0.868 ± 0.019	0.875 ± 0.030	0.862 ± 0.020	0.858 ± 0.019	all
						Poly-SVM	**0.987 ± 0.004**	**0.983 ± 0.010**	**0.991 ± 0.007**	**0.986 ± 0.005**	all
						RBF-SVM	**0.987 ± 0.004**	**0.983 ± 0.010**	0.990 ± 0.008	**0.985 ± 0.004**	all
						Random Forests	**0.987 ± 0.004**	**0.982 ± 0.011**	**0.991 ± 0.007**	**0.986 ± 0.004**	408.8 ± 570.1
						$CART_b$	**0.987 ± 0.005**	**0.984 ± 0.010**	0.990 ± 0.007	**0.986 ± 0.005**	1.0 ± 0.0
						$CART_{cv}$	**0.987 ± 0.005**	**0.983 ± 0.011**	0.990 ± 0.007	**0.985 ± 0.006**	1.6 ± 1.6
						SCM_b	**0.987 ± 0.005**	**0.984 ± 0.010**	0.990 ± 0.007	**0.986 ± 0.005**	1.0 ± 0.0
						SCM_{cv}	**0.987 ± 0.005**	**0.983 ± 0.010**	0.990 ± 0.007	**0.986 ± 0.005**	1.9 ± 0.6
	oxacillin	85	39	46	6.1	L1-logistic	0.988 ± 0.025	0.980 ± 0.043	**1.000 ± 0.000**	0.989 ± 0.023	97.6 ± 47.0
						L2-logistic	0.988 ± 0.025	0.980 ± 0.043	**1.000 ± 0.000**	0.989 ± 0.023	all*
						Majority	0.465 ± 0.131	0.100 ± 0.316	0.900 ± 0.316	–	–
						Naive Bayes	0.635 ± 0.072	0.777 ± 0.136	0.500 ± 0.153	0.658 ± 0.091	all
						Poly-SVM	0.988 ± 0.025	0.980 ± 0.043	**1.000 ± 0.000**	0.989 ± 0.023	all
						RBF-SVM	**1.000 ± 0.000**	**1.000 ± 0.000**	**1.000 ± 0.000**	**1.000 ± 0.000**	all
						Random Forests	0.988 ± 0.025	0.980 ± 0.043	**1.000 ± 0.000**	0.989 ± 0.023	15.9 ± 3.0

Continued on next page

Table S1. (Continued)

Species	Antibiotic	Genomes	Resistant	Susceptible	*k*-mers (millions)	Method	Accuracy	Sensitivity	Specificity	F1 score	Complexity
						CART$_b$	0.988 ± 0.025	0.980 ± 0.043	**1.000 ± 0.000**	0.989 ± 0.023	1.0 ± 0.0
						CART$_{cv}$	0.988 ± 0.025	0.980 ± 0.043	**1.000 ± 0.000**	0.989 ± 0.023	1.0 ± 0.0
						SCM$_b$	0.988 ± 0.025	0.980 ± 0.043	**1.000 ± 0.000**	0.989 ± 0.023	1.0 ± 0.0
						SCM$_{cv}$	0.988 ± 0.025	0.980 ± 0.043	**1.000 ± 0.000**	0.989 ± 0.023	1.0 ± 0.0
	penicillin	1042	886	156	12.1	L1-logistic	**0.974 ± 0.013**	0.981 ± 0.010	**0.934 ± 0.051**	**0.985 ± 0.008**	178881.5 ± 306100.4
						L2-logistic	**0.976 ± 0.011**	0.984 ± 0.009	**0.931 ± 0.055**	**0.986 ± 0.007**	all*
						Majority	0.853 ± 0.022	**1.000 ± 0.000**	0.000 ± 0.000	0.921 ± 0.013	–
						Naive Bayes	0.518 ± 0.041	0.468 ± 0.045	0.817 ± 0.059	0.622 ± 0.039	all
						Poly-SVM	**0.980 ± 0.011**	0.990 ± 0.007	0.923 ± 0.047	**0.988 ± 0.007**	all
						RBF-SVM	**0.977 ± 0.013**	0.988 ± 0.010	0.916 ± 0.052	**0.986 ± 0.008**	all
						Random Forests	**0.976 ± 0.011**	0.985 ± 0.007	**0.927 ± 0.051**	**0.986 ± 0.007**	4354.3 ± 7268.1
						CART$_b$	**0.973 ± 0.011**	0.980 ± 0.007	**0.934 ± 0.051**	**0.984 ± 0.007**	1.7 ± 0.5
						CART$_{cv}$	**0.971 ± 0.011**	0.979 ± 0.010	**0.930 ± 0.047**	**0.983 ± 0.007**	2.5 ± 0.7
						SCM$_b$	**0.975 ± 0.012**	0.983 ± 0.007	**0.927 ± 0.057**	**0.985 ± 0.007**	1.7 ± 0.5
						SCM$_{cv}$	**0.975 ± 0.012**	0.985 ± 0.006	0.920 ± 0.056	**0.985 ± 0.007**	2.5 ± 1.0
	tetracycline	1232	203	1029	12.3	L1-logistic	**0.986 ± 0.005**	**0.966 ± 0.029**	**0.991 ± 0.005**	**0.961 ± 0.015**	78129.4 ± 175973.3
						L2-logistic	**0.986 ± 0.006**	**0.957 ± 0.034**	**0.992 ± 0.006**	**0.960 ± 0.017**	all*
						Majority	0.820 ± 0.012	0.000 ± 0.000	**1.000 ± 0.000**	–	–
						Naive Bayes	0.919 ± 0.012	0.774 ± 0.075	0.951 ± 0.011	0.773 ± 0.044	all
						Poly-SVM	**0.982 ± 0.007**	0.942 ± 0.045	**0.991 ± 0.005**	0.949 ± 0.022	all
						RBF-SVM	**0.983 ± 0.008**	0.946 ± 0.044	**0.991 ± 0.006**	0.952 ± 0.023	all
						Random Forests	**0.987 ± 0.006**	**0.964 ± 0.031**	**0.993 ± 0.007**	**0.965 ± 0.019**	1572.5 ± 3038.9
						CART$_b$	**0.986 ± 0.005**	**0.966 ± 0.022**	**0.991 ± 0.005**	**0.961 ± 0.015**	2.0 ± 0.0
						CART$_{cv}$	**0.986 ± 0.005**	**0.966 ± 0.022**	**0.991 ± 0.005**	**0.961 ± 0.015**	2.0 ± 0.0
						SCM$_b$	**0.986 ± 0.005**	**0.966 ± 0.022**	**0.991 ± 0.005**	**0.961 ± 0.015**	2.0 ± 0.0
						SCM$_{cv}$	**0.986 ± 0.005**	**0.966 ± 0.022**	**0.991 ± 0.005**	**0.961 ± 0.015**	2.0 ± 0.0
	trimethoprim/sulfamethoxazole	320	142	178	6.9	L1-logistic	0.947 ± 0.025	0.889 ± 0.052	0.987 ± 0.018	0.931 ± 0.035	43517.4 ± 92826.5
						L2-logistic	**0.950 ± 0.022**	0.901 ± 0.049	0.985 ± 0.021	0.935 ± 0.034	all*
						Majority	0.578 ± 0.054	0.000 ± 0.000	**1.000 ± 0.000**	–	–
						Naive Bayes	0.928 ± 0.038	**0.916 ± 0.038**	0.936 ± 0.046	0.913 ± 0.049	all
						Poly-SVM	0.945 ± 0.025	0.889 ± 0.052	0.984 ± 0.022	0.930 ± 0.035	all
						RBF-SVM	0.941 ± 0.027	0.889 ± 0.052	0.977 ± 0.029	0.923 ± 0.039	all
						Random Forests	**0.956 ± 0.019**	0.901 ± 0.050	**0.995 ± 0.011**	**0.943 ± 0.026**	218.3 ± 415.7
						CART$_b$	**0.959 ± 0.020**	0.901 ± 0.050	**1.000 ± 0.000**	**0.947 ± 0.027**	1.0 ± 0.0
						CART$_{cv}$	**0.959 ± 0.020**	0.901 ± 0.050	**1.000 ± 0.000**	**0.947 ± 0.027**	1.0 ± 0.0
						SCM$_b$	**0.959 ± 0.020**	0.901 ± 0.050	**1.000 ± 0.000**	**0.947 ± 0.027**	1.0 ± 0.0
						SCM$_{cv}$	**0.959 ± 0.020**	0.901 ± 0.050	**1.000 ± 0.000**	**0.947 ± 0.027**	1.3 ± 0.9
S. enterica	ampicillin	347	279	68		L1-logistic	0.875 ± 0.041	0.914 ± 0.042	0.741 ± 0.172	0.920 ± 0.028	836.0 ± 972.0
						L2-logistic	0.913 ± 0.026	0.940 ± 0.030	0.822 ± 0.098	**0.944 ± 0.018**	all*
						Majority	0.791 ± 0.034	**1.000 ± 0.000**	0.000 ± 0.000	0.883 ± 0.022	–
						Naive Bayes	0.817 ± 0.038	0.969 ± 0.017	0.246 ± 0.113	0.893 ± 0.023	all
						Poly-SVM	0.909 ± 0.033	0.971 ± 0.020	0.685 ± 0.112	**0.943 ± 0.021**	all
						RBF-SVM	0.907 ± 0.031	0.973 ± 0.018	0.672 ± 0.111	**0.943 ± 0.020**	all
						Random Forests	0.910 ± 0.037	0.967 ± 0.014	0.707 ± 0.152	**0.944 ± 0.023**	3816.2 ± 4902.6

Continued on next page

Table S1. (Continued)

Species	Antibiotic	Genomes	Resistant	Susceptible	*k*-mers (millions)	Method	Accuracy	Sensitivity	Specificity	F1 score	Complexity
						CART$_b$	0.894±0.041	0.919±0.043	0.803±0.165	0.932±0.028	1.5 ± 0.8
						CART$_{cv}$	**0.925±0.039**	0.945±0.037	**0.855±0.087**	**0.951±0.027**	6.1 ± 3.1
						SCM$_b$	0.881±0.037	0.912±0.037	0.769±0.199	0.924±0.025	1.4 ± 0.5
						SCM$_{cv}$	**0.920±0.040**	0.950±0.040	0.808±0.089	**0.949±0.027**	5.5 ± 1.6
	chloramphenicol		251	96		L1-logistic	**0.925±0.039**	0.953±0.023	**0.867±0.107**	**0.946±0.030**	991.2 ± 1463.9
						L2-logistic	**0.929±0.033**	0.959±0.021	**0.864±0.102**	**0.950±0.026**	all*
						Majority	0.709±0.054	**1.000±0.000**	0.000±0.000	0.828±0.037	–
						Naive Bayes	0.759±0.053	**0.992±0.011**	0.198±0.092	0.853±0.036	all
						Poly-SVM	**0.920±0.030**	0.970±0.035	0.808±0.053	**0.944±0.023**	all
						RBF-SVM	**0.928±0.031**	0.976±0.024	0.822±0.084	**0.949±0.023**	all
						Random Forests	**0.926±0.029**	0.984±0.019	0.791±0.066	**0.949±0.022**	2354.8 ± 2780.1
						CART$_b$	0.913±0.024	0.943±0.035	0.848±0.067	0.938±0.020	1.0 ± 0.0
						CART$_{cv}$	0.900±0.045	0.961±0.032	0.761±0.108	0.931±0.033	3.6 ± 1.3
						SCM$_b$	0.913±0.024	0.943±0.035	0.848±0.067	0.938±0.020	1.0 ± 0.0
						SCM$_{cv}$	0.907±0.025	0.941±0.035	0.834±0.089	0.934±0.020	1.6 ± 1.3
	nalidixic acid		35	312		L1-logistic	**0.978±0.014**	**0.849±0.129**	**0.994±0.008**	**0.876±0.078**	181.0 ± 42.4
						L2-logistic	0.943±0.029	0.622±0.233	0.981±0.019	0.659±0.175	all*
						Majority	0.906±0.031	0.000±0.000	**1.000±0.000**	–	–
						Naive Bayes	0.893±0.034	0.049±0.087	0.981±0.018	–	all
						Poly-SVM	0.938±0.034	0.456±0.269	**0.991±0.011**	–	all
						RBF-SVM	0.942±0.029	0.474±0.220	**0.994±0.008**	0.592±0.201	all
						Random Forests	0.949±0.024	0.589±0.206	**0.991±0.008**	0.674±0.136	1871.7 ± 3412.6
						CART$_b$	**0.978±0.014**	**0.849±0.129**	**0.994±0.008**	**0.876±0.078**	1.0 ± 0.0
						CART$_{cv}$	**0.978±0.014**	**0.849±0.129**	**0.994±0.008**	**0.876±0.078**	1.0 ± 0.0
						SCM$_b$	**0.978±0.014**	**0.849±0.129**	**0.994±0.008**	**0.876±0.078**	1.0 ± 0.0
						SCM$_{cv}$	**0.978±0.014**	**0.849±0.129**	**0.994±0.008**	**0.876±0.078**	1.0 ± 0.0
	spectinomycin	290	233	57	5.6	L1-logistic	0.890±0.028	0.959±0.028	**0.629±0.086**	0.932±0.018	4557.6 ± 3948.5
						L2-logistic	0.886±0.023	0.959±0.021	0.618±0.110	0.930±0.015	all*
						Majority	0.791±0.034	**1.000±0.000**	0.000±0.000	0.883±0.022	–
						Naive Bayes	0.850±0.051	0.987±0.018	0.341±0.170	0.912±0.030	all
						Poly-SVM	0.893±0.028	0.983±0.020	0.555±0.101	0.935±0.018	all
						RBF-SVM	0.888±0.023	0.972±0.022	0.576±0.073	0.932±0.015	all
						Random Forests	**0.912±0.033**	**0.993±0.011**	0.607±0.118	**0.947±0.021**	87.8 ± 93.7
						CART$_b$	**0.919±0.026**	**0.996±0.009**	**0.629±0.102**	**0.951±0.016**	1.0 ± 0.0
						CART$_{cv}$	**0.917±0.023**	**0.994±0.010**	**0.629±0.102**	**0.950±0.014**	1.4 ± 1.0
						SCM$_b$	**0.919±0.026**	**0.996±0.009**	**0.629±0.102**	**0.951±0.016**	1.0 ± 0.0
						SCM$_{cv}$	**0.917±0.024**	**0.991±0.011**	**0.638±0.106**	**0.950±0.015**	1.9 ± 1.6
	streptomycin	347	291	56	6.9	L1-logistic	0.943±0.026	0.970±0.029	0.826±0.075	**0.965±0.016**	90.3 ± 21.2
						L2-logistic	0.929±0.032	0.959±0.038	0.791±0.062	0.956±0.021	all*
						Majority	0.822±0.033	**1.000±0.000**	0.000±0.000	0.902±0.020	–
						Naive Bayes	0.842±0.032	0.975±0.027	0.224±0.073	0.910±0.020	all
						Poly-SVM	0.925±0.044	0.982±0.029	0.665±0.156	0.955±0.026	all
						RBF-SVM	0.925±0.042	0.981±0.030	0.668±0.139	0.955±0.025	all
						Random Forests	0.938±0.031	0.972±0.028	0.785±0.135	0.962±0.019	258.2 ± 370.7
						CART$_b$	0.943±0.026	0.970±0.029	0.828±0.069	**0.965±0.016**	1.1 ± 0.3

Continued on next page

Table S1. (Continued)

Species	Antibiotic	Genomes	Resistant	Susceptible	k-mers (millions)	Method	Accuracy	Sensitivity	Specificity	F1 score	Complexity
						CART$_{cv}$	**0.946 ± 0.030**	0.975 ± 0.030	0.818 ± 0.076	**0.967 ± 0.019**	3.3 ± 2.9
						SCM$_b$	**0.946 ± 0.027**	0.970 ± 0.029	**0.839 ± 0.067**	**0.967 ± 0.017**	1.1 ± 0.3
						SCM$_{cv}$	**0.954 ± 0.028**	0.980 ± 0.030	**0.831 ± 0.073**	**0.972 ± 0.018**	1.9 ± 0.6
	sulphonamides	341	306	35	5.8	L1-logistic	**0.946 ± 0.017**	0.987 ± 0.013	0.607 ± 0.120	**0.970 ± 0.010**	16480.8 ± 24362.6
						L2-logistic	**0.951 ± 0.017**	0.990 ± 0.015	0.643 ± 0.136	**0.973 ± 0.010**	all*
						Majority	0.891 ± 0.033	**1.000 ± 0.000**	0.000 ± 0.000	0.942 ± 0.019	–
						Naive Bayes	0.878 ± 0.033	0.964 ± 0.019	0.191 ± 0.149	0.933 ± 0.019	all
						Poly-SVM	0.943 ± 0.022	**0.993 ± 0.008**	0.540 ± 0.138	**0.968 ± 0.013**	all
						RBF-SVM	0.931 ± 0.032	0.989 ± 0.013	0.480 ± 0.158	0.962 ± 0.018	all
						Random Forests	**0.954 ± 0.015**	0.990 ± 0.011	**0.657 ± 0.165**	**0.975 ± 0.008**	2614.9 ± 3500.2
						CART$_b$	0.909 ± 0.021	0.973 ± 0.033	0.406 ± 0.243	0.950 ± 0.011	1.6 ± 0.5
						CART$_{cv}$	0.918 ± 0.026	0.981 ± 0.019	0.419 ± 0.222	0.955 ± 0.014	3.0 ± 1.4
						SCM$_b$	0.913 ± 0.022	0.976 ± 0.032	0.447 ± 0.263	0.952 ± 0.012	1.6 ± 0.7
						SCM$_{cv}$	0.931 ± 0.021	0.984 ± 0.016	0.492 ± 0.236	0.962 ± 0.011	2.4 ± 0.7
	tetracycline	347	280	67	6.9	L1-logistic	0.888 ± 0.036	0.923 ± 0.042	0.769 ± 0.148	0.929 ± 0.025	1806.7 ± 1360.8
						L2-logistic	0.914 ± 0.032	0.949 ± 0.022	0.789 ± 0.095	0.946 ± 0.021	all*
						Majority	0.793 ± 0.034	**1.000 ± 0.000**	0.000 ± 0.000	0.884 ± 0.022	–
						Naive Bayes	0.790 ± 0.042	0.980 ± 0.026	0.064 ± 0.064	0.880 ± 0.027	all
						Poly-SVM	**0.935 ± 0.016**	0.971 ± 0.015	0.799 ± 0.086	**0.959 ± 0.010**	all
						RBF-SVM	**0.933 ± 0.020**	0.978 ± 0.014	0.767 ± 0.068	**0.959 ± 0.013**	all
						Random Forests	0.912 ± 0.041	0.958 ± 0.024	0.739 ± 0.147	0.945 ± 0.025	2740.5 ± 2676.5
						CART$_b$	0.910 ± 0.028	0.921 ± 0.019	**0.877 ± 0.093**	0.942 ± 0.020	2.2 ± 0.6
						CART$_{cv}$	0.909 ± 0.037	0.945 ± 0.032	0.778 ± 0.110	0.942 ± 0.025	6.6 ± 3.2
						SCM$_b$	0.912 ± 0.036	0.923 ± 0.029	**0.877 ± 0.100**	0.943 ± 0.025	2.0 ± 0.0
						SCM$_{cv}$	0.906 ± 0.030	0.938 ± 0.028	0.799 ± 0.143	0.940 ± 0.021	3.6 ± 1.4
	trimethoprim	341	45	296	5.8	L1-logistic	0.916 ± 0.029	0.510 ± 0.232	0.969 ± 0.032	0.555 ± 0.178	109872.9 ± 152324.6
						L2-logistic	0.921 ± 0.027	**0.544 ± 0.235**	0.971 ± 0.027	0.585 ± 0.159	all*
						Majority	0.887 ± 0.033	0.000 ± 0.000	**1.000 ± 0.000**	–	–
						Naive Bayes	0.871 ± 0.040	0.220 ± 0.128	0.954 ± 0.034	–	all
						Poly-SVM	**0.931 ± 0.024**	0.466 ± 0.155	0.990 ± 0.009	0.588 ± 0.135	all
						RBF-SVM	**0.928 ± 0.026**	0.458 ± 0.172	0.988 ± 0.011	0.574 ± 0.145	all
						Random Forests	**0.928 ± 0.025**	0.435 ± 0.189	0.990 ± 0.009	0.555 ± 0.168	1091.7 ± 2035.5
						CART$_b$	**0.937 ± 0.029**	0.497 ± 0.188	**0.993 ± 0.011**	**0.626 ± 0.176**	1.0 ± 0.0
						CART$_{cv}$	**0.929 ± 0.032**	0.531 ± 0.187	0.980 ± 0.025	**0.617 ± 0.188**	2.9 ± 2.1
						SCM$_b$	**0.937 ± 0.029**	0.497 ± 0.188	**0.993 ± 0.011**	**0.626 ± 0.176**	1.0 ± 0.0
						SCM$_{cv}$	**0.929 ± 0.032**	0.531 ± 0.187	0.980 ± 0.025	**0.617 ± 0.188**	1.7 ± 1.3
S. haemolyticus	ciprofloxacin	120	74	46	5.3	L1-logistic	**0.925 ± 0.047**	0.955 ± 0.052	**0.883 ± 0.102**	**0.938 ± 0.042**	279.1 ± 616.8
						L2-logistic	0.838 ± 0.057	0.894 ± 0.080	0.778 ± 0.167	0.867 ± 0.060	all*
						Majority	0.629 ± 0.126	**1.000 ± 0.000**	0.000 ± 0.000	0.765 ± 0.103	–
						Naive Bayes	0.758 ± 0.136	0.678 ± 0.216	**0.892 ± 0.103**	0.756 ± 0.172	all
						Poly-SVM	0.829 ± 0.077	0.856 ± 0.059	0.794 ± 0.196	0.859 ± 0.067	all
						RBF-SVM	0.846 ± 0.068	0.877 ± 0.082	0.810 ± 0.117	0.871 ± 0.067	all
						Random Forests	0.846 ± 0.040	0.903 ± 0.075	0.783 ± 0.137	0.875 ± 0.042	2820.0 ± 3407.8
						CART$_b$	**0.925 ± 0.047**	0.955 ± 0.052	**0.883 ± 0.102**	**0.938 ± 0.042**	1.0 ± 0.0
						CART$_{cv}$	**0.933 ± 0.053**	0.961 ± 0.054	**0.892 ± 0.109**	**0.944 ± 0.046**	1.0 ± 0.0

Continued on next page

Table S1. (Continued)

Species	Antibiotic	Genomes	Resistant	Susceptible	k-mers (millions)	Method	Accuracy	Sensitivity	Specificity	F1 score	Complexity
						SCM$_b$	**0.925±0.047**	0.955±0.052	**0.883±0.102**	**0.938±0.042**	1.0 ± 0.0
						SCM$_{cv}$	**0.933±0.053**	0.961±0.054	**0.892±0.109**	**0.944±0.046**	1.0 ± 0.0
	fusidic acid	114	39	75	5.2	L1-logistic	**0.832±0.113**	0.729±0.222	0.879±0.107	**0.749±0.172**	2732.4 ± 1611.9
						L2-logistic	0.786±0.105	0.716±0.181	0.825±0.134	0.704±0.132	all*
						Majority	0.636±0.091	0.000±0.000	**1.000±0.000**	–	–
						Naive Bayes	0.800±0.084	**0.742±0.117**	0.821±0.102	0.725±0.107	all
						Poly-SVM	0.800±0.123	0.702±0.174	0.855±0.132	0.715±0.155	all
						RBF-SVM	0.809±0.100	0.675±0.175	0.890±0.084	0.714±0.125	all
						Random Forests	0.818±0.132	0.723±0.186	0.866±0.125	**0.744±0.182**	1158.0 ± 2277.1
						CART$_b$	**0.827±0.113**	**0.743±0.241**	0.872±0.093	**0.743±0.181**	1.8 ± 0.4
						CART$_{cv}$	0.773±0.117	0.662±0.243	0.820±0.120	0.664±0.199	2.0 ± 0.7
						SCM$_b$	**0.827±0.109**	0.700±0.256	0.893±0.107	0.728±0.182	1.7 ± 0.5
						SCM$_{cv}$	0.782±0.137	0.654±0.273	0.845±0.096	0.664±0.233	2.5 ± 1.4
	tetracycline	100	37	63	5.1	L1-logistic	0.780±0.067	0.669±0.107	0.853±0.082	0.698±0.064	1550.3 ± 1271.7
						L2-logistic	**0.810±0.061**	0.744±0.102	0.856±0.074	**0.745±0.073**	all*
						Majority	0.620±0.082	0.000±0.000	**1.000±0.000**	–	–
						Naive Bayes	0.780±0.079	**0.809±0.155**	0.769±0.136	0.731±0.101	all
						Poly-SVM	0.745±0.093	0.574±0.259	0.874±0.091	0.600±0.187	all
						RBF-SVM	0.750±0.085	0.584±0.244	0.874±0.091	0.613±0.171	all
						Random Forests	0.795±0.055	0.623±0.191	0.917±0.075	0.685±0.108	1071.9 ± 1233.9
						CART$_b$	0.785±0.082	0.635±0.163	0.892±0.106	0.688±0.112	1.0 ± 0.0
						CART$_{cv}$	0.735±0.088	0.686±0.174	0.772±0.156	0.658±0.087	2.7 ± 1.9
						SCM$_b$	0.770±0.116	0.603±0.155	0.885±0.159	0.667±0.139	1.0 ± 0.0
						SCM$_{cv}$	0.730±0.075	0.561±0.128	0.838±0.075	0.606±0.084	2.2 ± 0.9
S. pneumoniae	cefuroxime	113	68	45	5.7	L1-logistic	**0.977±0.039**	0.983±0.038	0.966±0.060	**0.979±0.038**	777.9 ± 1049.9
						L2-logistic	0.932±0.069	0.934±0.060	0.947±0.088	0.938±0.072	all*
						Majority	0.618±0.127	**1.000±0.000**	0.000±0.000	0.757±0.104	–
						Naive Bayes	0.877±0.080	0.807±0.164	0.978±0.049	0.875±0.112	all
						Poly-SVM	0.900±0.082	0.894±0.102	0.931±0.092	0.911±0.079	all
						RBF-SVM	0.891±0.084	0.879±0.113	0.937±0.087	0.901±0.083	all
						Random Forests	**0.986±0.031**	0.983±0.038	**0.992±0.024**	**0.986±0.035**	290.2 ± 691.2
						CART$_b$	0.945±0.052	0.976±0.050	0.903±0.079	0.951±0.050	1.0 ± 0.0
						CART$_{cv}$	0.941±0.043	0.969±0.052	0.897±0.084	0.947±0.046	1.0 ± 0.0
						SCM$_b$	0.945±0.052	0.976±0.050	0.903±0.079	0.951±0.050	1.0 ± 0.0
						SCM$_{cv}$	0.936±0.038	0.956±0.051	0.911±0.089	0.944±0.043	1.2 ± 0.4
	chloramphenicol	409	149	260	6.4	L1-logistic	0.948±0.022	**0.950±0.023**	0.947±0.036	0.927±0.031	1391.5 ± 1844.4
						L2-logistic	0.949±0.020	0.936±0.029	0.957±0.028	0.928±0.027	all*
						Majority	0.654±0.036	0.000±0.000	**1.000±0.000**	–	–
						Naive Bayes	0.910±0.013	0.936±0.034	0.896±0.017	0.877±0.020	all
						Poly-SVM	0.946±0.021	0.929±0.039	0.955±0.027	0.922±0.030	all
						RBF-SVM	0.944±0.023	0.925±0.041	0.955±0.031	0.920±0.032	all
						Random Forests	**0.957±0.019**	**0.947±0.030**	0.962±0.026	**0.938±0.027**	92.2 ± 110.8
						CART$_b$	**0.960±0.018**	**0.951±0.035**	0.966±0.026	**0.943±0.026**	1.0 ± 0.0
						CART$_{cv}$	**0.959±0.018**	**0.947±0.035**	0.966±0.026	**0.941±0.026**	1.0 ± 0.0
						SCM$_b$	**0.960±0.018**	**0.951±0.035**	0.966±0.026	**0.943±0.026**	1.0 ± 0.0

Continued on next page

Table S1. (Continued)

Species	Antibiotic	Genomes	Resistant	Susceptible	k-mers (millions)	Method	Accuracy	Sensitivity	Specificity	F1 score	Complexity
						SCM$_{cv}$	**0.959**$\pm$**0.018**	**0.947**$\pm$**0.035**	0.966$\pm$0.026	**0.941**$\pm$**0.026**	1.0 $\pm$ 0.0
	clindamycin	145	28	117	6.0	L1-logistic	**0.986**$\pm$**0.024**	**0.950**$\pm$**0.127**	**0.996**$\pm$**0.013**	0.959$\pm$0.082	211.2 $\pm$ 208.2
						L2-logistic	0.948$\pm$0.034	0.833$\pm$0.187	0.979$\pm$0.022	0.842$\pm$0.112	all*
						Majority	0.810$\pm$0.080	0.000$\pm$0.000	**1.000**$\pm$**0.000**	–	–
						Naive Bayes	0.907$\pm$0.046	0.809$\pm$0.233	0.932$\pm$0.045	0.735$\pm$0.192	all
						Poly-SVM	0.938$\pm$0.048	0.760$\pm$0.292	0.979$\pm$0.022	–	all
						RBF-SVM	0.945$\pm$0.037	0.780$\pm$0.236	0.984$\pm$0.021	0.802$\pm$0.190	all
						Random Forests	**0.986**$\pm$**0.024**	**0.950**$\pm$**0.127**	**0.996**$\pm$**0.014**	**0.962**$\pm$**0.079**	225.7 $\pm$ 532.5
						CART$_b$	**0.990**$\pm$**0.023**	**0.950**$\pm$**0.127**	**1.000**$\pm$**0.000**	**0.970**$\pm$**0.079**	1.0 $\pm$ 0.0
						CART$_{cv}$	**0.990**$\pm$**0.023**	**0.950**$\pm$**0.127**	**1.000**$\pm$**0.000**	**0.970**$\pm$**0.079**	1.0 $\pm$ 0.0
						SCM$_b$	**0.990**$\pm$**0.023**	**0.950**$\pm$**0.127**	**1.000**$\pm$**0.000**	**0.970**$\pm$**0.079**	1.0 $\pm$ 0.0
						SCM$_{cv}$	**0.990**$\pm$**0.023**	**0.950**$\pm$**0.127**	**1.000**$\pm$**0.000**	**0.970**$\pm$**0.079**	1.0 $\pm$ 0.0
	erythromycin	324	247	77	6.3	L1-logistic	**0.961**$\pm$**0.028**	0.970$\pm$0.023	0.932$\pm$0.086	**0.974**$\pm$**0.019**	4386.0 $\pm$ 4378.0
						L2-logistic	0.948$\pm$0.029	0.966$\pm$0.033	0.897$\pm$0.075	**0.965**$\pm$**0.020**	all*
						Majority	0.742$\pm$0.047	**1.000**$\pm$**0.000**	0.000$\pm$0.000	0.851$\pm$0.031	–
						Naive Bayes	0.706$\pm$0.034	0.716$\pm$0.034	0.686$\pm$0.133	0.783$\pm$0.029	all
						Poly-SVM	0.941$\pm$0.030	0.964$\pm$0.034	0.872$\pm$0.077	0.960$\pm$0.022	all
						RBF-SVM	0.941$\pm$0.032	0.962$\pm$0.035	0.879$\pm$0.069	0.960$\pm$0.023	all
						Random Forests	0.934$\pm$0.040	0.976$\pm$0.022	0.823$\pm$0.111	0.956$\pm$0.028	4155.2 $\pm$ 5134.4
						CART$_b$	**0.952**$\pm$**0.026**	0.951$\pm$0.027	**0.950**$\pm$**0.078**	**0.966**$\pm$**0.019**	2.2 $\pm$ 0.4
						CART$_{cv}$	0.948$\pm$0.027	0.951$\pm$0.031	0.937$\pm$0.080	0.964$\pm$0.019	3.1 $\pm$ 1.1
						SCM$_b$	**0.952**$\pm$**0.026**	0.951$\pm$0.027	**0.950**$\pm$**0.078**	**0.966**$\pm$**0.019**	2.2 $\pm$ 0.4
						SCM$_{cv}$	0.950$\pm$0.030	0.959$\pm$0.024	0.920$\pm$0.096	**0.966**$\pm$**0.021**	2.8 $\pm$ 0.8
	meropenem	114	32	82	5.8	L1-logistic	**0.864**$\pm$**0.074**	**0.907**$\pm$**0.172**	0.851$\pm$0.122	**0.783**$\pm$**0.140**	411.8 $\pm$ 470.4
						L2-logistic	**0.868**$\pm$**0.079**	0.812$\pm$0.164	0.888$\pm$0.075	0.762$\pm$0.201	all*
						Majority	0.705$\pm$0.086	0.000$\pm$0.000	**1.000**$\pm$**0.000**	–	–
						Naive Bayes	0.836$\pm$0.089	0.818$\pm$0.192	0.839$\pm$0.071	0.721$\pm$0.214	all
						Poly-SVM	0.818$\pm$0.068	0.676$\pm$0.184	0.883$\pm$0.077	0.666$\pm$0.178	all
						RBF-SVM	0.818$\pm$0.068	0.676$\pm$0.184	0.883$\pm$0.077	0.666$\pm$0.178	all
						Random Forests	0.850$\pm$0.074	0.799$\pm$0.166	0.893$\pm$0.100	0.742$\pm$0.154	313.2 $\pm$ 209.0
						CART$_b$	0.850$\pm$0.077	**0.914**$\pm$**0.101**	0.827$\pm$0.111	0.769$\pm$0.150	1.0 $\pm$ 0.0
						CART$_{cv}$	0.827$\pm$0.093	0.733$\pm$0.240	0.850$\pm$0.117	0.685$\pm$0.220	2.4 $\pm$ 1.4
						SCM$_b$	**0.864**$\pm$**0.091**	0.876$\pm$0.166	0.846$\pm$0.121	0.771$\pm$0.205	1.0 $\pm$ 0.0
						SCM$_{cv}$	0.850$\pm$0.088	0.860$\pm$0.178	0.832$\pm$0.108	0.750$\pm$0.204	1.2 $\pm$ 0.4
	penicillin	172	113	59		L1-logistic	**0.994**$\pm$**0.012**	**0.996**$\pm$**0.014**	**0.992**$\pm$**0.024**	**0.995**$\pm$**0.010**	233.3 $\pm$ 219.9
						L2-logistic	0.976$\pm$0.033	0.988$\pm$0.027	0.953$\pm$0.062	0.983$\pm$0.024	all*
						Majority	0.694$\pm$0.068	**1.000**$\pm$**0.000**	0.000$\pm$0.000	0.818$\pm$0.048	–
						Naive Bayes	0.835$\pm$0.048	0.774$\pm$0.066	0.970$\pm$0.049	0.865$\pm$0.045	all
						Poly-SVM	0.941$\pm$0.046	0.964$\pm$0.044	0.889$\pm$0.094	0.958$\pm$0.031	all
						RBF-SVM	0.950$\pm$0.042	0.975$\pm$0.035	0.889$\pm$0.094	0.964$\pm$0.029	all
						Random Forests	**0.994**$\pm$**0.012**	**0.996**$\pm$**0.014**	**0.986**$\pm$**0.045**	**0.996**$\pm$**0.009**	436.0 $\pm$ 730.2
						CART$_b$	0.982$\pm$0.025	**0.996**$\pm$**0.014**	0.949$\pm$0.073	**0.988**$\pm$**0.018**	1.0 $\pm$ 0.0
						CART$_{cv}$	0.982$\pm$0.025	**0.996**$\pm$**0.014**	0.949$\pm$0.073	**0.988**$\pm$**0.018**	1.0 $\pm$ 0.0
						SCM$_b$	0.982$\pm$0.025	**0.996**$\pm$**0.014**	0.949$\pm$0.073	**0.988**$\pm$**0.018**	1.0 $\pm$ 0.0
						SCM$_{cv}$	0.979$\pm$0.020	0.983$\pm$0.021	0.969$\pm$0.053	0.985$\pm$0.014	1.0 $\pm$ 0.0

Continued on next page

Table S1. (Continued)

Species	Antibiotic	Genomes	Resistant	Susceptible	*k*-mers (millions)	Method	Accuracy	Sensitivity	Specificity	F1 score	Complexity
	tetracycline	393	284	109	6.2	L1-logistic	0.956 ± 0.020	0.976 ± 0.015	0.909 ± 0.042	0.969 ± 0.015	9330.3 ± 9714.3
						L2-logistic	0.956 ± 0.022	0.978 ± 0.012	0.902 ± 0.050	0.969 ± 0.016	all*
						Majority	0.714 ± 0.050	**1.000 ± 0.000**	0.000 ± 0.000	0.832 ± 0.035	–
						Naive Bayes	0.869 ± 0.057	0.887 ± 0.067	0.822 ± 0.057	0.904 ± 0.047	all
						Poly-SVM	0.949 ± 0.018	0.980 ± 0.015	0.874 ± 0.047	0.964 ± 0.013	all
						RBF-SVM	0.942 ± 0.017	0.973 ± 0.012	0.869 ± 0.050	0.960 ± 0.013	all
						Random Forests	0.959 ± 0.025	0.985 ± 0.014	0.894 ± 0.057	**0.971 ± 0.017**	493.3 ± 840.0
						$CART_b$	**0.964 ± 0.019**	0.985 ± 0.014	0.911 ± 0.042	**0.975 ± 0.013**	1.0 ± 0.0
						$CART_{cv}$	**0.965 ± 0.019**	0.977 ± 0.020	**0.937 ± 0.037**	**0.976 ± 0.014**	2.6 ± 1.6
						SCM_b	**0.964 ± 0.019**	0.985 ± 0.014	0.911 ± 0.042	**0.975 ± 0.013**	1.0 ± 0.0
						SCM_{cv}	**0.971 ± 0.016**	0.983 ± 0.016	**0.937 ± 0.037**	**0.979 ± 0.012**	2.4 ± 0.8
	trimethoprim/sulfamethoxazole	2826	2187	639	24.2	L1-logistic	0.928 ± 0.011	0.942 ± 0.014	**0.880 ± 0.024**	0.953 ± 0.008	7172.4 ± 6532.7
						L2-logistic	0.926 ± 0.010	0.943 ± 0.019	0.867 ± 0.030	0.952 ± 0.008	all*
						Majority	0.778 ± 0.015	**1.000 ± 0.000**	0.000 ± 0.000	0.875 ± 0.009	–
						Naive Bayes	0.854 ± 0.019	0.852 ± 0.026	0.858 ± 0.032	0.900 ± 0.015	all
						Poly-SVM	**0.935 ± 0.006**	0.969 ± 0.008	0.815 ± 0.041	**0.958 ± 0.004**	all
						RBF-SVM	**0.934 ± 0.006**	0.970 ± 0.009	0.811 ± 0.042	**0.958 ± 0.004**	all
						Random Forests	**0.943 ± 0.008**	0.984 ± 0.005	0.801 ± 0.025	**0.964 ± 0.005**	19693.3 ± 25788.2
						$CART_b$	**0.939 ± 0.010**	0.973 ± 0.008	0.819 ± 0.029	**0.961 ± 0.007**	5.4 ± 1.0
						$CART_{cv}$	**0.938 ± 0.010**	0.971 ± 0.009	0.822 ± 0.030	**0.960 ± 0.007**	8.6 ± 4.4
						SCM_b	**0.938 ± 0.011**	0.981 ± 0.013	0.789 ± 0.017	**0.961 ± 0.007**	3.1 ± 0.3
						SCM_{cv}	**0.937 ± 0.008**	0.983 ± 0.006	0.778 ± 0.024	**0.961 ± 0.005**	4.0 ± 2.8

Table S2. Extended benchmark. Comparison to state-of-the-art classifiers in terms of accuracy and model complexity. For each dataset the accuracy is shown, along with the number of k-mers used by the model (in parentheses). Results are shown for Set Covering Machines (SCM), Classification trees (CART), Random Forests[13] with χ^2 feature selection, Logistic regression with L1 and L2 regularization and χ^2 feature selection (L1-logistic, L2-logistic), Polynomial kernel and RBF kernel Support Vector Machines (Poly-SVM, RBF-SVM), Naive Bayes, and a baseline predictor that predicts the most abundant class in the data (Majority). Accuracies within 1% of the maximum value are shown in bold. Results are averaged over ten repetitions of the experiment.

Dataset	SCM_b	$CART_b$	Random forests*, †	L1-logistic*, †	L2-logistic*, †	Poly-SVM†	RBF-SVM†	Naive Bayes	Majority
A. baumannii	0.849 (2.7)	0.864 (3.4)	**0.892** (6314.6)	0.880 (3980.5)	**0.885** (1e6)	**0.886** (all)	0.880 (all)	0.822 (all)	0.644
E. coli	**0.818** (4.6)	**0.808** (7.0)	**0.812** (39289.6)	0.792 (3727.2)	0.789 (1e6)	0.779 (all)	0.776 (all)	0.634 (all)	0.697
E. faecium	**1.000** (1.0)	**1.000** (1.0)	**1.000** (202.6)	**1.000** (142.0)	**1.000** (1e6)	**0.996** (all)	**0.992** (all)	0.808 (all)	0.588
K. pneumoniae	**0.950** (3.9)	**0.949** (4.3)	**0.956** (42856.8)	**0.952** (7607.4)	**0.948** (1e6)	0.943 (all)	0.943 (all)	0.760 (all)	0.571
M. tuberculosis	**0.963** (4.5)	**0.962** (4.7)	**0.962** (78761.3)	**0.962** (2242.2)	0.941 (1e6)	0.934 (all)	0.930 (all)	0.789 (all)	0.658
N. gonorrhoeae	**0.935** (3.0)	**0.936** (3.3)	0.895 (4571.7)	**0.942** (6095.6)	0.915 (1e6)	0.906 (all)	0.905 (all)	0.736 (all)	0.529
P. aeruginosa	**0.939** (1.2)	**0.942** (1.1)	0.874 (21600.5)	**0.937** (87.8)	0.828 (1e6)	0.773 (all)	0.762 (all)	0.768 (all)	0.588
P. difficile	**0.982** (1.0)	**0.982** (1.0)	0.949 (662.2)	0.957 (121.8)	0.936 (1e6)	0.949 (all)	0.951 (all)	0.887 (all)	0.599
S. aureus	**0.987** (1.0)	**0.987** (1.0)	**0.987** (408.8)	**0.988** (230.6)	**0.987** (1e6)	**0.987** (all)	**0.987** (all)	0.868 (all)	0.544
S. enterica	0.913 (1.0)	0.913 (1.0)	**0.926** (2354.8)	**0.925** (991.2)	**0.929** (1e6)	**0.920** (all)	**0.928** (all)	0.759 (all)	0.709
S. haemolyticus	**0.925** (1.0)	**0.925** (1.0)	0.846 (2820.0)	**0.925** (279.1)	0.838 (1e6)	0.829 (all)	0.846 (all)	0.758 (all)	0.629
S. pneumoniae	**0.960** (1.0)	**0.960** (1.0)	**0.957** (92.2)	0.948 (1391.5)	0.949 (1e6)	0.946 (all)	0.944 (all)	0.910 (all)	0.654

* For scalability reasons, these algorithms were trained using feature selection to select the one million k-mers that were most associated with the phenotypes; all other k-mers were discarded (see *Methods*).

† The implementations available in Scikit-Learn 0.18.2 were used. When applicable, the kernel matrices were precomputed using custom code based on the k-mer matrices.

Table S3. Sample compression bound values for the SCM_b models on each benchmark dataset. We report other quantities that are relevant to the interpretation of the bound, such as the accuracy and complexity (number of rules) of the models and the number of examples in the datasets. We also show the number of k-mers in each dataset, since, surprisingly, this value does not take part in the calculation of the bound. The reader is encouraged to observe the expression of the bound (Equation (2)) in parallel to understand how each quantity affects the bound. Note that values that varied over ten repetitions of the experiment are shown as mean $\pm$ standard deviation.

Dataset	Bound	Accuracy	Complexity	Examples	k-mers (millions)
A. baumannii	0.427 ± 0.022	0.849 ± 0.031	2.7 ± 0.5	499	42.4
E. coli	0.473 ± 0.007	0.818 ± 0.019	4.6 ± 1.1	1524	48.5
E. faecium	0.236 ± 0.000	1.000 ± 0.000	1.0 ± 0.0	134	10.3
K. pneumoniae	0.220 ± 0.003	0.950 ± 0.007	3.9 ± 0.7	2107	70.3
M. tuberculosis	0.160 ± 0.003	0.963 ± 0.005	4.5 ± 0.5	5022	11.7
N. gonorrhoeae	0.331 ± 0.018	0.935 ± 0.030	3.0 ± 0.0	392	4.8
P. aeruginosa	0.284 ± 0.015	0.939 ± 0.023	1.2 ± 0.4	491	43.0
P. difficile	0.164 ± 0.009	0.982 ± 0.009	1.0 ± 0.0	462	19.8
S. aureus	0.074 ± 0.005	0.987 ± 0.005	1.0 ± 0.0	1593	13.3
S. enterica	0.369 ± 0.012	0.913 ± 0.024	1.0 ± 0.0	347	6.9
S. haemolyticus	0.431 ± 0.027	0.925 ± 0.047	1.0 ± 0.0	120	5.3
S. pneumoniae	0.233 ± 0.013	0.960 ± 0.018	1.0 ± 0.0	409	6.4

Table S4. Sample compression bound values for the $CART_b$ models on each benchmark dataset. We report other quantities that are relevant to the interpretation of the bound, such as the accuracy and complexity (number of rules) of the models and the number of examples in the datasets. We also show the number of k-mers in each dataset, since, surprisingly, this value does not take part in the calculation of the bound. The reader is encouraged to observe the expression of the bound (Equation (3)) in parallel to understand how each quantity affects the bound. Note that values that varied over ten repetitions of the experiment are shown as mean ± standard deviation.

Dataset	Bound	Accuracy	Complexity	Examples	k-mers (millions)
A. baumannii	0.423 ± 0.010	0.864 ± 0.042	3.4 ± 0.7	499	42.4
E. coli	0.464 ± 0.008	0.808 ± 0.021	7.0 ± 0.7	1524	48.5
E. faecium	0.249 ± 0.000	1.000 ± 0.000	1.0 ± 0.0	134	10.3
K. pneumoniae	0.223 ± 0.004	0.949 ± 0.007	4.3 ± 1.2	2107	70.3
M. tuberculosis	0.162 ± 0.003	0.962 ± 0.004	4.7 ± 1.2	5022	11.7
N. gonorrhoeae	0.341 ± 0.017	0.936 ± 0.039	3.3 ± 0.5	392	4.8
P. aeruginosa	0.288 ± 0.015	0.942 ± 0.028	1.1 ± 0.3	491	43.0
P. difficile	0.168 ± 0.009	0.982 ± 0.009	1.0 ± 0.0	462	19.8
S. aureus	0.076 ± 0.005	0.987 ± 0.005	1.0 ± 0.0	1593	13.3
S. enterica	0.373 ± 0.012	0.913 ± 0.024	1.0 ± 0.0	347	6.9
S. haemolyticus	0.442 ± 0.027	0.925 ± 0.047	1.0 ± 0.0	120	5.3
S. pneumoniae	0.237 ± 0.013	0.960 ± 0.018	1.0 ± 0.0	409	6.4

References

1. Littlestone, N. & Warmuth, M. Relating data compression and learnability. Tech. Rep., University of California Santa Cruz, Santa Cruz, CA (1986).
2. Floyd, S. & Warmuth, M. Sample compression, learnability, and the Vapnik-Chervonenkis dimension. *Mach. Learn.* **21**, 269–304 (1995).
3. Marchand, M. & Sokolova, M. Learning with decision lists of data-dependent features. *J. Mach. Learn. Res.* **6**, 427–451 (2005).
4. Vapnik, V. *Statistical learning theory. 1998*, vol. 3 (Wiley, New York, 1998).
5. Mendelson, S. Rademacher averages and phase transitions in glivenko-cantelli classes. *IEEE transactions on Inf. Theory* **48**, 251–263 (2002).
6. Yıldız, O. T. Vc-dimension of univariate decision trees. *IEEE transactions on neural networks learning systems* **26**, 378–387 (2015).
7. Bartlett, P. L. & Mendelson, S. Rademacher and gaussian complexities: Risk bounds and structural results. *J. Mach. Learn. Res.* **3**, 463–482 (2002).
8. Kääriäinen, M., Malinen, T. & Elomaa, T. Selective rademacher penalization and reduced error pruning of decision trees. *J. Mach. Learn. Res.* **5**, 1107–1126 (2004).
9. Drouin, A. *et al.* Predictive computational phenotyping and biomarker discovery using reference-free genome comparisons. *BMC genomics* **17**, 754 (2016).
10. Marchand, M. & Shawe-Taylor, J. The set covering machine. *The J. Mach. Learn. Res.* **3**, 723–746 (2002).
11. Shah, M. Sample compression bounds for decision trees. In *Proceedings of the 24th international conference on Machine learning*, 799–806 (ACM, 2007).
12. Breiman, L., Friedman, J., Stone, C. J. & Olshen, R. A. *Classification and regression trees* (CRC press, New York, 1984).
13. Breiman, L. Random forests. *Mach. learning* **45**, 5–32 (2001).
